# Exploring the Landscape of the PP7 Virus-like Particle for Peptide Display

**DOI:** 10.1021/acsnano.3c06178

**Published:** 2023-09-05

**Authors:** Parisa Keshavarz-Joud, Liangjun Zhao, Daija Bobe, Carolina Hernandez, Mykhailo Kopylov, Laura Y. Yen, Naima Djeddar, Brianna Thompson, Caleb Connors, Greg Gibson, Anton Bryksin, M.G. Finn

**Affiliations:** † School of Chemistry and Biochemistry, Georgia Institute of Technology, Atlanta, Georgia 30306, United States; ‡ New York Structural Biology Center, New York, New York 10027, United States; § Parker H. Petit Institute for Bioengineering and Biosciences, Georgia Institute of Technology, Atlanta, Georgia 30306, United States; ∥ School of Biological Sciences, Georgia Institute of Technology, Atlanta, Georgia 30306, United States

**Keywords:** virus-like particles, peptide display, molecular library, selection, self-assembly

## Abstract

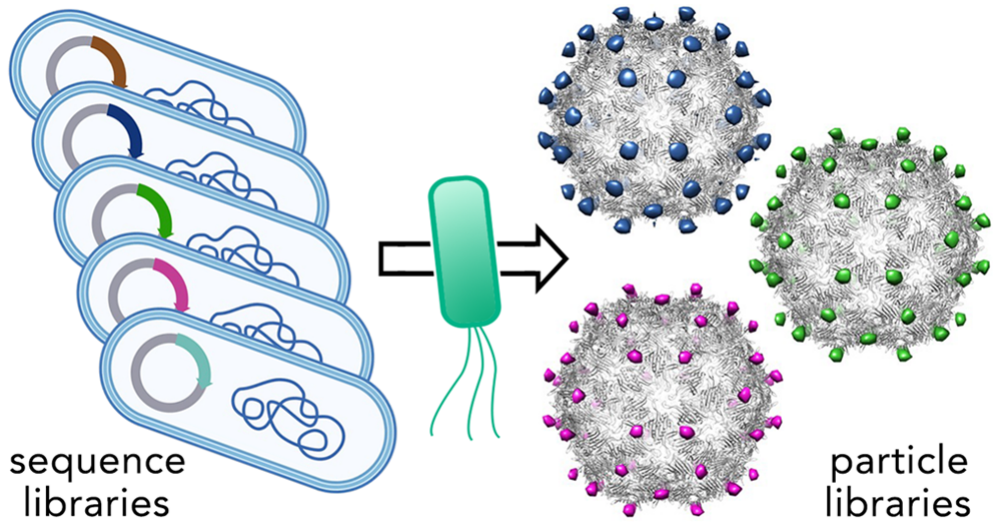

Self-assembling virus-like particles (VLPs) can tolerate a wide degree of genetic and chemical manipulation to their capsid protein to display a foreign molecule polyvalently. We previously reported the successful incorporation of foreign peptide sequences in the junction loop and onto the C-terminus of PP7 dimer VLPs, as these regions are accessible for surface display on assembled capsids. Here, we report the implementation of a library-based approach to test the assembly tolerance of PP7 dimer capsid proteins to insertions or terminal extensions of randomized 15-mer peptide sequences. By performing two iterative rounds of assembly-based selection, we evaluated the degree of favorability of all 20 amino acids at each of the 15 randomized positions. Deep sequencing analysis revealed a distinct preference for the inclusion of hydrophilic peptides and negatively charged amino acids (Asp and Glu) and the exclusion of positively charged peptides and bulky and hydrophobic amino acid residues (Trp, Phe, Tyr, and Cys). Within the libraries tested here, we identified 4000 to 22,000 unique 15-mer peptide sequences that can successfully be displayed on the surface of the PP7 dimer capsid. Overall, the use of small initial libraries consisting of no more than a few million members yielded a significantly larger number of unique and assembly-competent VLP sequences than have been previously characterized for this class of nucleoprotein particle.

## Introduction

Virus-like particles (VLPs) are self-assembled nanoscale protein cages derived from naturally occurring viruses, lacking a natural genome for replication. VLP assembly typically requires no external guidance, such as from molecular chaperones, giving the investigator ready access to multivalent structures for a myriad of uses. Many VLPs can be recombinantly expressed in high yields and are amenable to genetic and chemical modifications to display functional molecules on the exterior surface. Especially popular in this regard are capsids derived from the Leviviridae RNA bacteriophages,^1^ including MS2,^2,3^ Qβ,^4,5^ and PP7.^6,7^ In addition, various methods have been developed to encapsulate molecular payloads inside VLPs for imaging, catalysis, and delivery.^8−13^ Important for the subject of this paper is the natural tendency of most VLPs to encapsulate RNA randomly or in directed fashion^10^ from the expression host, usually *E. coli*.

The most common applications of VLPs often employ them as polyvalent display platforms for molecular entities that engage target materials, cells, or tissues. The attachment of such entities can be accomplished by a variety of bioconjugation techniques.^14^ Peptide or protein sequences are often attached by methods that require polypeptide adapters such as SpyTag/SpyCatcher^15,16^ or enzyme-catalyzed processes such as sortase ligation.^17−19^

For the desired display of polypeptides, it would be most efficient if the desired sequences were simply added or inserted into the coding sequence for the coat (or capsid) protein (CP), as long as added amino acids do not disrupt the particle assembly. In our experience with Qβ^20,21^ and PP7,^22^ we have found PP7VLPs to be more tolerant of C-terminal extensions of various sizes. This may be because the C-termini of the PP7 coat proteins are farther away from each other than in Qβ, making C-terminal extensions less crowded and therefore more likely to fold or assemble properly. If such genomic additions are successfully incorporated, then resulting particles have the added peptide present in every subunit.^22^ This provides the most uniform display possible of the desired structure. However, not all point mutations, insertions, and extensions are tolerated, and what determines success or failure remains elusive.

Several attempts have been made to incorporate random peptide sequences into MS2^23^ and PP7,^7^ but these were limited to single-point mutations or short peptide additions. Peabody and colleagues studied the effect of 6-, 8-, and 10-mer sequences added to the externally exposed AB loops of MS2 and PP7. They compared the tolerance of random peptide library insertions in the wild-type (WT) and “single-chain dimer” cyclic peptides, the latter constructed from genetic fusion of the C-terminal end of one CP to the N-terminal of another with a dipeptide (Tyr-Gly) linker. The study showed that a single-chain dimer CP is generally more amenable to genetic modifications in both MS2 and PP7; however, the overall analysis of allowed sequences and effects on particle assembly was quite limited. From each library, fewer than 25 colonies of transformants were handpicked for pooled expression, and the sequences of assembly-competent peptides were not identified.

Tullman-Ercek and colleagues have taken an effective approach to address three different needs in virus-like particle display using mutational libraries and next-generation sequencing to cover more sequence space. First, they challenged MS2 self-assembly by incorporating single-point mutations individually at every position, discovering a particle that is significantly more acid-sensitive than the native MS2 capsid.^24^ They have also assessed the fitness landscape of the particle to randomized 3-mer peptide N-terminal extensions^25^ or loop insertions.^26^ In the former, they significantly enhanced the ability of the MS2 particle to tolerate additions to the N-termini of the capsid proteins (showing a surprising effect of positively charged residues). In the latter, high-throughput sequencing of the resulting assembled particles identified physical features, such as charge, hydrogen bonding, and flexibility of the introduced foreign peptides. Chackerian and colleagues recently demonstrated an important use of VLP libraries by creating a family of MS2 particles each displaying multiple copies of an overlapping series of linear peptides from the Zika virus and used this pool to characterize the immune response of Zika-infected patients.^27^

While VLPs derived from viruses such as MS2 and PP7 do not contain viral genomic material, studies of mutational VLP libraries depend on the packaging of polynucleotide coding for the protein sequence to establish a connection between genotype and phenotype. Tullman-Ercek and colleagues took advantage of the ability of the MS2 capsid to act as carriers for mRNA encoding the coat protein sequence by virtue of random entrainment during expression and assembly in the *E. coli* production host;^24^ the interior surfaces of all of the Leviviridae particles are highly positively charged.^28^ We similarly found that PP7VLPs isolated from a standard expression culture contained RNA that included the entire capsid protein sequence, established by disruption of the isolated particle, isolation of the released RNA, reverse transcription, and DNA sequencing. In some constructs, we also appended the RNA stem-loop that naturally directs gene packaging^29−31^ to the coat protein sequence to increase its packaging efficiency, as we have described previously for Qβ.^10^ In all cases, although we do not expect every particle to contain the desired mRNA sequence, packaging proved to be efficient enough to conduct informative mutational explorations.

Here we describe our first use of genomic libraries to assess the foreign peptide fusion tolerance landscape of the PP7 virus-like particle, focusing on longer (15-mer) extensions and insertions to allow for a wider diversity of properties and to relate more directly to the display of antigenic or cell-targeting motifs. In this study, we made no effort to cover a significant portion of the possible sequence space but rather focused on sampling representative libraries of 10^5^–10^6^ members. These small libraries produced unexpected diversities of assembled particle structures and several general trends that begin to define rules for VLP design.

## Results and Discussion

We chose single-chain PP7 dimer VLPs as our platform for this work, as we^22^ and others^7^ have found them to be more tolerant to genetic modifications compared to the WT construct. The starting coat protein dimer variant was described previously,^22^ consisting of two copies of PP7 CPs connected with a tetrapeptide AYGG linker. From this platform, we created two types of randomized libraries using standard cloning techniques to explore the tolerance of the PP7 dimer VLP to peptide fusions: 15 amino acid extensions (connected by the constant octapeptide GGASESGA, designed to provide a flexible spacer) to the C-terminus of the dimeric protein and 15 amino acid insertions between the two glycine residues in the bridge between subunits (AYG-15mer-G). PP7, like other bacteriophages, naturally encapsulates its single-stranded RNA genome; the VLP entrains mixtures of RNA via charge-based interactions with the interior surface of the capsid. In the past, we have included a capsid-binding RNA hairpin to enhance packaging of desired oligonucleotides and associated proteins.^10^ As noted in the experimental details, this was done for some, but not all, of the libraries used here; we found that the positively charged interior of the capsid protein is sufficient for packaging its negatively charged coding mRNA, with enough of the relevant mRNA to establish the necessary linkage between genotype and phenotype.

### Library Cloning and Preliminary Characterization

The PP7 dimer VLP libraries with loop insertion or C-terminal extensions were constructed using synthetic oligonucleotides (Table S1) containing either NNS or VNS codons (where N = A, T, C, or G; S = C or G; V = A, C, or G) to encode the randomized peptide sequences. NNS codons allow for the expression of all 20 natural amino acids and the TAG stop codon, while the VNS construct excludes the hydrophobic amino acids (Cys, Phe, Trp, and Tyr) and the stop codons. The synthetic oligonucleotide templates were ordered at 200 nmol scale to provide a maximum possible library size of 1.2 × 10^17^ sequences, vastly under-sampling the possible sequence space: approximately 0.00032% of all possible 15-mer NNS combinations [(4 × 4 × 2)^15^ = 3.8 × 10^22^] and 0.024% of all possible 15-mer VNS combinations [(3 × 4 × 2)^15^ = 5.0 × 10^20^]. All oligonucleotide libraries included constant 5′ and 3′ flanking regions to enable polymerase chain reaction (PCR) amplification prior to subcloning into codon-optimized expression vectors and provide handles for generating amplicons for next-generation sequencing (NGS) (Table S1).

Transformation of ligated plasmids into NEB-5α chemically competent *E. coli* cells was performed with efficiencies of 10^5^–10^6^ cfu/αμg, assuming complete prior ligation of the vector with library fragments. Transformed libraries were analyzed by inoculation and growth of an overnight culture, extracting DNA from the collected cells, and Sanger sequencing, confirming insertion of the randomized 15-mer into the CP template in all cases (Figure S1) before NGS. While the presence of small amounts of parental vector could not be excluded by this analysis, this vector would be incapable of producing assembled PP7VLPs as it encodes superfolder green fluorescence protein (sfGFP) in the intended library position (Figure S2), with this fusion preventing VLP assembly. Therefore, the presence of trace amounts of the parental vector would be unlikely to significantly influence the assembly-based selection process. The plasmid library samples passing the Sanger sequencing test were used for the VLP library expression and purification. Transformation using electrocompetent BL21(DE3) *E. coli* cells resulted in 10^6^–10^7^ cfu as the maximum library size of expressed CP sequences.

### Selection of Assembly-Competent PP7 Dimer Variants

**Figure 1:**
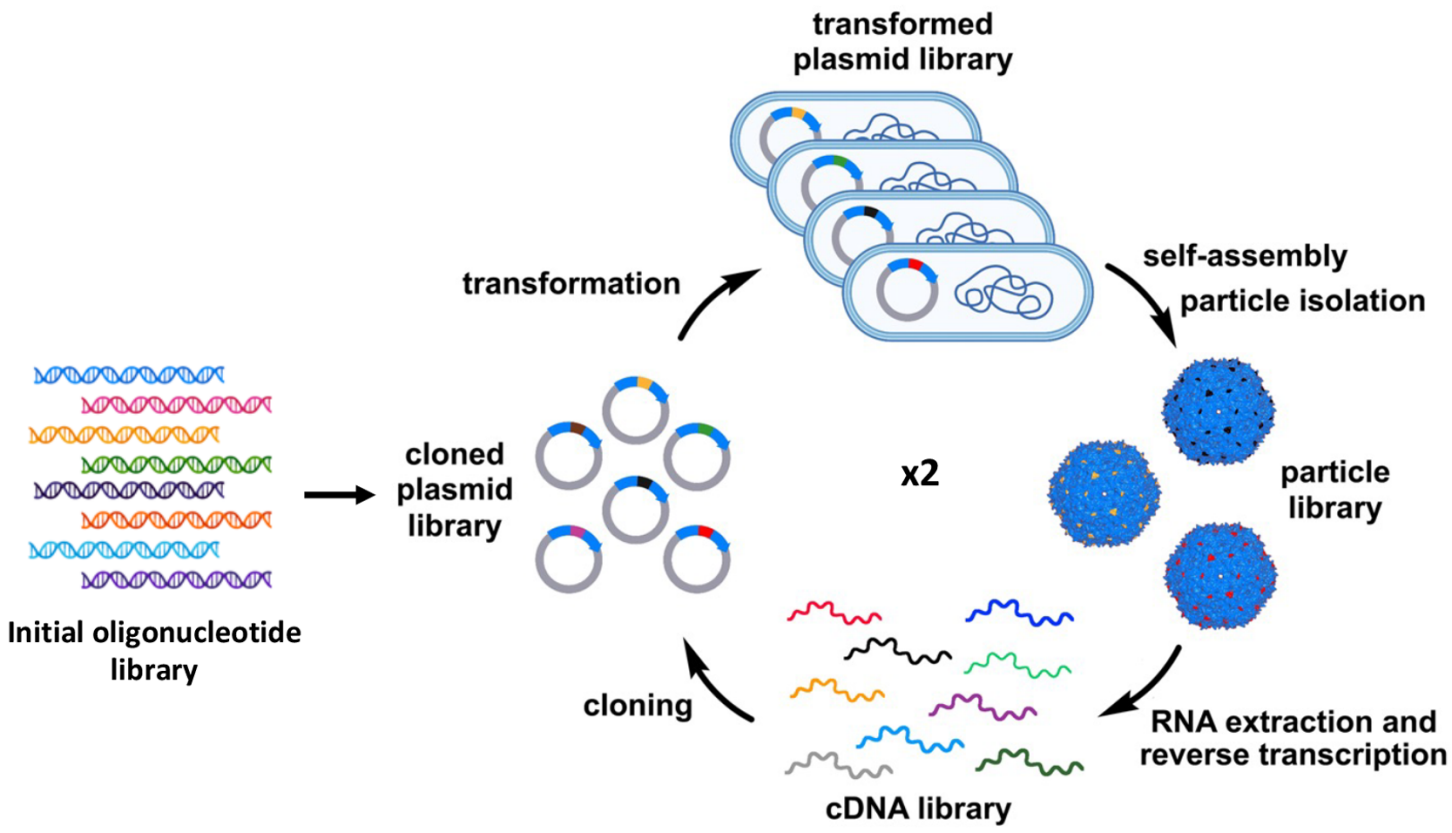
Assembly-based selection of PP7 virus-like particles bearing randomized loop insertions or C-terminal peptide extensions.

The assembly-based selection was performed, as illustrated in Figure 1. After IPTG induction, particle expression and assembly were allowed to proceed for 16–18 h at room temperature. The assembled VLPs were isolated from the bacterial culture as discussed in the Experimental Section. The yield of the isolated assembled VLPs was determined, and particles were characterized by (a) negative stain transmission electron microscopy (TEM), (b) denaturation and mass spectrometry, (c) dynamic light scattering (DLS), (d) size-exclusion chromatography (SEC), and (e) next-generation sequencing of cDNA generated from the RNA recovered from the isolated particles. The cDNA was also PCR amplified and used to create a second-generation plasmid library (without further sequence randomization) that was taken through the same assembly-based selection cycle, including protein expression, particle isolation, and analysis. In all, eight libraries were generated: four initial (generation 1, or G1) and four enriched (generation 2, or G2) libraries coding for 15-mer loop insertions or 15-mer C-terminal extensions using NNS or VNS codon sets, as follows.

**Figure 2:**
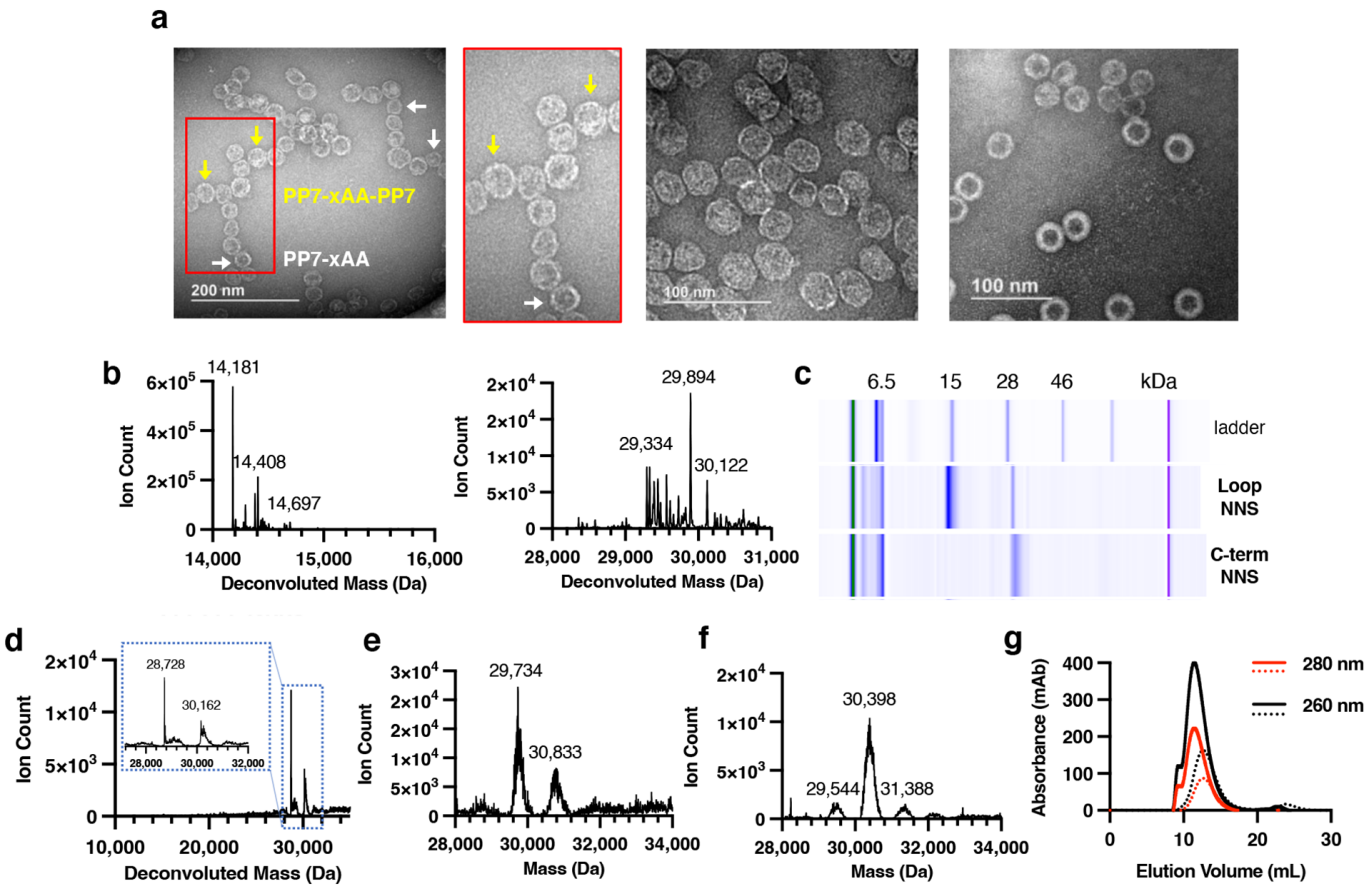
Representative analyses of particles isolated from loop insert and C-terminal extension PP7 libraries. (a) TEM images: (left, with expanded section) G1 loop NNS library with monomeric and dimeric VLPs labeled in white and yellow arrows, respectively; (middle) G1 C-term NNS extension library; (right) G1 loop VNS library. (b) Mass distributions of particles isolated from the G1 loop NNS library with the mass ranges of monomeric CPs (left) and dimeric CPs (right), shown separately. (c) Microfluidic gel electrophoresis analysis of G1 loop and C-term NNS libraries. (d–f) Mass distributions of particles isolated from G1 C-term NNS and close-up of the 28–32 kDa mass range, G1 loop VNS (e), and G1 C-term VNS (f) libraries. (g) SEC trace of the G1 loop VNS library (in solid lines) compared to wild-type PP7VLP (dotted lines).

TEM analysis of G1 loop NNS library samples (Figure 2a) showed two distinct populations: smaller particles with an average diameter of 27.7 nm (white arrows) and larger particles with an average diameter of 34.5 nm (yellow arrows). This was expected based on the potential for the TAG stop codon to be included in the loop sequence (with a calculated random-chance probability of 38%, since the stop codon has a 1/32 chance of appearing at each position of the 15-mer). This results in the production of monomeric coat proteins with variable-length (1–14 amino acid) C-terminal extensions, all of which are expected to produce “wild-type” *T* = 3 particles of approximately 26.5 nm diameter containing 180 monomeric subunits.^32,33^ The larger particles represent the population of linked dimer-based structures with *T* = 4 symmetry and 120 dimeric subunits that were similar to the previously characterized four-amino-acid linker PP7-AYGG-PP7 with a diameter of 32.8 nm.^22^ The presence of both monomeric and dimeric PP7VLPs was verified by LC-MS analysis of denatured samples, which showed both monomeric (14–15 kDa) and dimeric (29–31 kDa) populations (Figure 2b). Furthermore, gel electrophoresis of denatured particles (Figure 2c) demonstrated the coexistence of both particle populations. Interestingly, despite statistical probabilities favoring the presence of dimeric sequences, there was noticeably a higher abundance of monomeric coat proteins compared to dimers.

The G1 C-term NNS library exhibited a greater degree of morphological homogeneity in TEM analysis, with an average diameter of 33.6 nm, similar to that of PP7-AYGG-PP7VLP (Figure 2a). Gel electrophoresis (Figure 2c) and mass spectra (Figure 2d) of this sample similarly only showed the presence of dimeric CPs. Interestingly, the observed *m*/*z* and peak intensity values did not reflect the expected probability of stop codon appearance in the C-term library. For example, an intense peak was observed at 28,728 Da, corresponding to the placement of the stop codon at the first NNS position, and two distinct mass distributions were observed rather than a continuous range. This likely reflects a difference in assembly competence, as the particles with no extended amino acids are more likely to assemble. It is also possible that unequal degrees of proteolytic degradation occur before or after assembly, but we regard this as unlikely since we see no evidence of such degradation when expressing single particles having such extensions. While we do not have enough data to refine these hypotheses, these results show that even small mutational libraries are subject to a variety of factors that contribute to fitness for production, assembly, and isolation.

VLPs assembled from the G1 loop VNS library displayed an average diameter of 35.4 nm (determined by TEM, Figure 2a) and CP masses of 28–32 kDa (Figure 2e) as expected for the exclusive production of PP7 dimer VLPs because of the elimination of the stop codon. A similar range of CP masses was observed for the G1 C-term VNS library (Figure 2f). Size-exclusion chromatography of the isolated particle ensemble further validated the homogeneity of the purified VLPs and the encapsulation of mRNA within the intact capsid particles (Figures 2g and S3). The yields of assembled particles from VNS libraries were roughly 5-fold greater than those from the first-generation NNS libraries (Table 1), suggesting that one or more of the amino acids omitted in VNS codons is unfavorable to expression or assembly.

**Table 1:** Assembled Particle Yields (from 1 L of *E. coli* Culture) and the Number of Total and Unique 15-mer Sequences (Appearing at Least Twice) of Particles Isolated from Each Library

generation	15-mer position	codon set	yield (mg)	# transform. sequence^a^	# unique transform. sequence^a^	average transform. count/unique sequence^a^	# cDNA sequences^b^	# unique cDNA sequence^b^	average cDNA count/unique sequence^b^
G1	loop	NNS	0.2	151,187	30,232	5.0	83,631	12,057	6.9
G2	loop	NNS	0.8	78,172	3,252	24.0	52,705	2,454	21.5
G1	C-term	NNS	0.6	74,461	23,336	3.2	14,145	4,064	3.5
G2	C-term	NNS	2.0	17,319	1,369	12.7	15,705	1,262	12.4
G1	loop	VNS	5.5	321,595	29,325	11.0	281,890	22,043	12.8
G2	loop	VNS	11.0	132,336	4,872	27.2	194,650	6,318	30.8
G1	C-term	VNS	1.5	62,443	22,016	2.8	49,894	15,793	3.2
G2	C-term	VNS	5.5	58,373	4,127	14.1	57,144	3,928	14.5

a From transformed *E. coli* cells (transformation libraries).

b From isolated particles (cDNA libraries).

The cDNA libraries from all four generation 1 (G1) constructs (G1 loop NNS, G1 C-term NNS, G1 loop VNS, and G1 C-term VNS) were PCR amplified, cloned back into the expression vector, and subjected to a second round of particle production and analysis, as depicted in Figure 1. To assess reproducibility and subcloning bias, we performed this enrichment operation on three independent samples of the G1 C-term NNS library, obtaining nearly identical results. In the second round, particle yields consistently increased by 2- to 4-fold compared to those in the first round (Table 1), confirming the selection of assembly-competent members of the initial transformation library. Particle sizes and coat protein mass distributions of the generation 2 (G2) particle libraries were similar to their respective G1, as measured by DLS, LC-MS, and SEC analyses (Figures S3–S5). TEM images of the G2 C-term NNS library indicated high levels of homogeneity with little variation in observed sizes and morphologies (average diameter of 35.3 nm; Figure S4e).

### Next-Generation Sequencing Analysis

To determine the sequences of the assembled particles, mRNA was extracted from the purified particles and promptly reverse transcribed into cDNA using oligo-(dT) primers. The variable 15-mer segment within each was then PCR amplified using upstream- and downstream-specific primers (Table S1) to add indices and library-specific barcodes. For comparison, the BL21(DE3) *E. coli* cells that were successfully transformed for VLP production were processed to isolate plasmid DNA (referred to here as the “transformed library” or “T”) and prepared for NGS, providing the starting pool of sequences for each assembly-based selection experiment (Figure 1). Assuming minimal sequence bias in these standard PCR operations, comparison of the cDNA library (also referred to as “C”) to the transformed library allows us to identify chemical or physical characteristics in the peptide sequence that promote or inhibit CP expression and self-assembly.

Sequencing of the G1 NNS transformed plasmid libraries revealed that 56–63% of the sequences within the loop insertion and C-terminal extension libraries contained the full 15-mer (Figure S6), matching the expected probability of omitting the TAG stop codon in each of the 15 positions: (31/32)^15^ = 62.1%. Aside from minimal negative bias toward Ile at position 1 and minimal positive bias toward Met at the same position, the transformed plasmid libraries exhibited the same codon frequencies as the expected NNS and VNS statistical distributions, thereby showing that cloning and transformation did not introduce any additional amino acid bias (Figure S7).

To evaluate the sequence diversity of the cDNA libraries obtained from assembled and isolated particles, we removed sequences that appeared only once in the NGS reads, constituting approximately 70–85% of the distinct peptide sequences. The resulting G1 cDNA libraries displayed varying sizes, ranging from approximately 4000 to 22,000 unique peptide sequences, as summarized in Table 1 and Figure S8. Notably, these relatively straightforward experiments, utilizing small initial libraries consisting of no more than a few million members, yielded a significantly larger number of unique and assembly-competent VLP sequences compared to previous characterizations.

**Figure 3:**
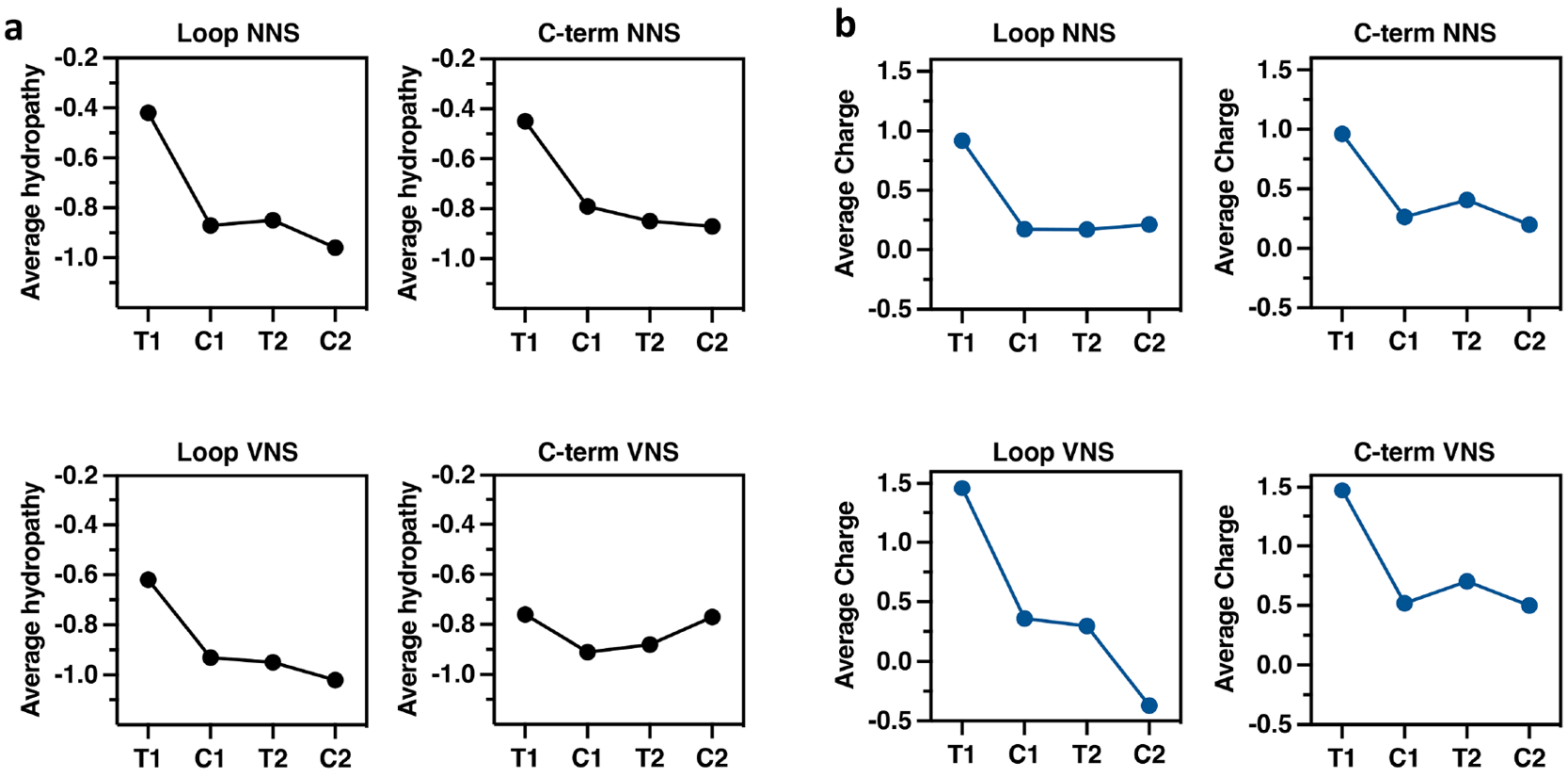
Average hydropathy (a) and charge (b) of 15-mer peptides in each VLP library. T1 = generation 1 transformation library (possible sequences expressed in *E. coli*), C1 = generation 1 cDNA library (particles recovered and sequenced), and T2 and C2 = transformation and cDNA libraries for generation 2, respectively.

**Figure 4:**
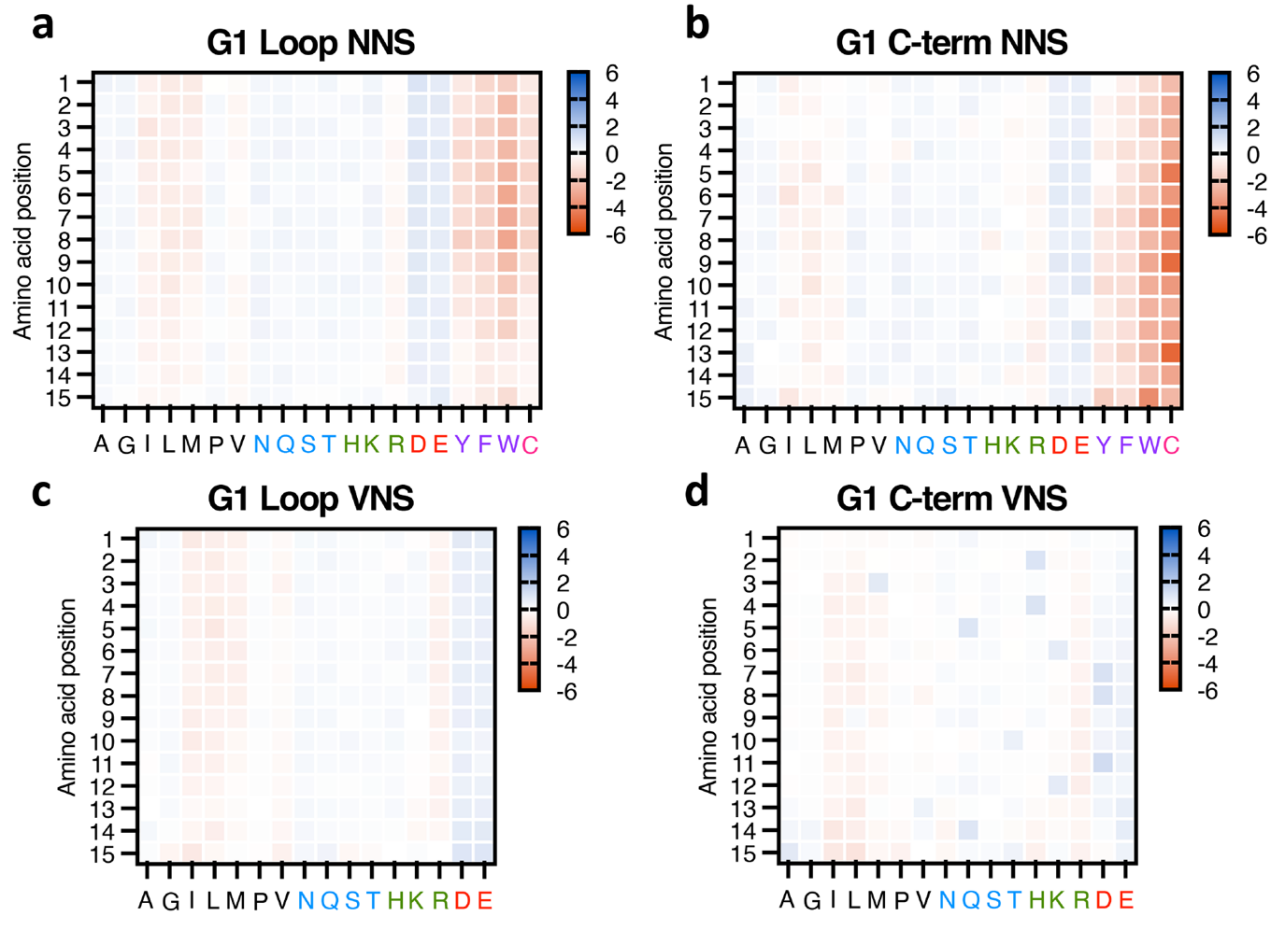
Favorability, measured by log_2_ of the abundance ratio (cDNA library/transformed library) of each amino acid at each position of the full 15-mer peptides. (a) Generation 1 (G1) loop NNS, (b) G1 C-term NNS, (c) G1 loop VNS, (d) G1 C-term VNS. Amino acids are grouped by their properties indicated by the color of the single-letter code (hydrophobic = black, polar/uncharged = blue, basic and positively charged = green, acidic and negatively charged = red, aromatic = purple, and thiol containing = pink). The data are plotted here on a common heat map scale to enable comparisons between libraries. For representations of the data using individual minimum and maximum values, see Figure S11.

Comparison of the chemical characteristics of the successfully displayed peptide sequences relative to those of the transformed library revealed several features that appear to favor successful particle assembly by capsid proteins bearing these peptides as loops and C-terminal extensions. First, the successfully displayed peptides were significantly more hydrophilic than the overall set of possible peptide sequences, as shown by negative shifts in average hydropathy scores (GRAVY index)^34^ going from the transformation to cDNA libraries in the initial (G1) selections, as shown in Figures 3 and S10a. The preference for hydrophilic surface residues is consistent with the need for particles to avoid hydrophobic interactions that may divert the coat proteins from proper folding and assembly and for the assembled particles to avoid excessive aggregation that can be damaging to the particle stability after assembly, as we have often observed when too many hydrophobic molecules are conjugated to polyvalent VLP surfaces. Thus, Phe, Tyr, and Trp experienced significant negative selection, and cysteine was strongly disfavored in these surface-exposed peptides, presumably because of its tendency to induce oxidative aggregation and therefore disappearance from collections of soluble particles (Figures 4a,b and S9).

The use of VNS libraries removed disfavored side chains, resulting in an increase in the number of unique peptide sequences (with frequency ≥2) appearing in the isolated VLPs by 1.8-fold (loop) and 3.9-fold (C-term) compared to the NNS constructs (Table 1 and Figure S8). A shift toward more hydrophilic residues in assembled particles (relative to the capsid protein sequences transformed into *E. coli*) was still observed for the loop insertions introduced by VNS codons but not for the C-terminal peptides (Figure 3a). Examination of relative amino acid incorporation (Figures 4 and S9) showed these libraries to be very similar in slightly negative selection against hydrophobic Leu, Ile, and Met, as well as cationic Arg, but compensation was observed in the C-terminal library by selection in favor of the polar Asp, His, Lys, and Gln at certain positions.

The assembled VLPs from both NNS and VNS libraries also displayed consistent and substantial shifts to lower overall charge in the inserted or appended peptides and a notable cutoff of positively charged 15-mer sequences (Figures 3b and S10b). This charge adjustment was accomplished mostly by enrichment in aspartate and glutamate residues by approximately 30–35%, as well as very mild selection against Arg; His and Lys were neither enriched nor diminished to significant degrees (Figure 4). [The discussion above refers to particles containing full-length 15-mer insertions or extensions. Of course, we also obtained assembled particles from G1 NNS library members containing stop codons in the randomized loop or extension peptides, giving rise to monomeric and dimeric coat protein particles, respectively, each containing a C-terminal extension of less than 15 amino acids. The same type of sequence analysis of these particles, summarized in Figure S12, revealed very similar patterns of amino acid favorability.]

To ensure the validity of our findings and rule out any bias in the oligonucleotide library or limitations of NGS, we conducted two additional replicates of the experiment using the G1 C-term NNS library (Figure S13). These replicates involved the independent synthesis of oligonucleotide libraries that were cloned at the 3′ end of the PP7 dimer coding region. Replicate 2 was sequenced using the MiSeq platform, while replicate 3 was sequenced using Illumina MiniSeq. Each replicate experiment showed different specific patterns of enrichment relative to the starting libraries, an expected outcome for vastly undersampled libraries wherein each replicate is assumed to contain mostly non-overlapping sets of starting sequences. However, the overall functional trends observed for all of the peptide libraries explored in this work were found to be reproducible. (1) The expected percentage of sequences containing the full 15-mer was consistent (56–71%, Figure S13c). (2) Hydrophilic, smaller, and negatively charged peptides were favored, with strong selection against Y, F, W, and C and weaker selection against I, L, and M (Figure S13b). (3) Average hydropathy and charge both became more negative in the assembled particles of each replicate (Figure S13d), although replicate 3 provided a unique variation in which histidine and arginine were enhanced at a few isolated positions, giving a truncated charge distribution (Figure S13e) and an overall smaller diminution in the overall peptide charge for the library (Figure S13d). Again, we regard this as a natural consequence of starting with undersampled sequence libraries. Note that, even though many more reads were generated for replicate 3 by the MiniSeq analysis (approximately 230,000, 8–16 times that of other replicates), the number of unique sequences was not much greater (5411, compared to 4064 and 2440 for replicates 1 and 2, respectively, Figure S13a). This suggests that most of the unique pool of assembly-competent VLPs was identified in each case.

Amplification and expression of the initial selected libraries provided much greater yields of particles in each case (Table 1), as expected for the enrichment of assembly- and isolation-competent sequences. However, with one exception, the generation-2 VLPs were not very different in calculated hydropathy or charge (Figures 3 and S14). The exception was the loop VNS library, which acquired sequences with more negative charge in the second selection step; at present, we do not know if this is a significant trend. In all G2 VLP libraries, while certain amino acid residues were either more or less favored, depending on their positions within the 15-mer (Figure S15), the average fold bias throughout the 15 possible positions converged to 0 (Figure S16). Additionally, the average frequency of unique 15-mers displayed on the PP7 dimer VLPs in the second generation increased by 2.4–4.6-fold compared to their respective first-generation constructs (Table 1).

### Morphological Analysis

**Figure 5:**
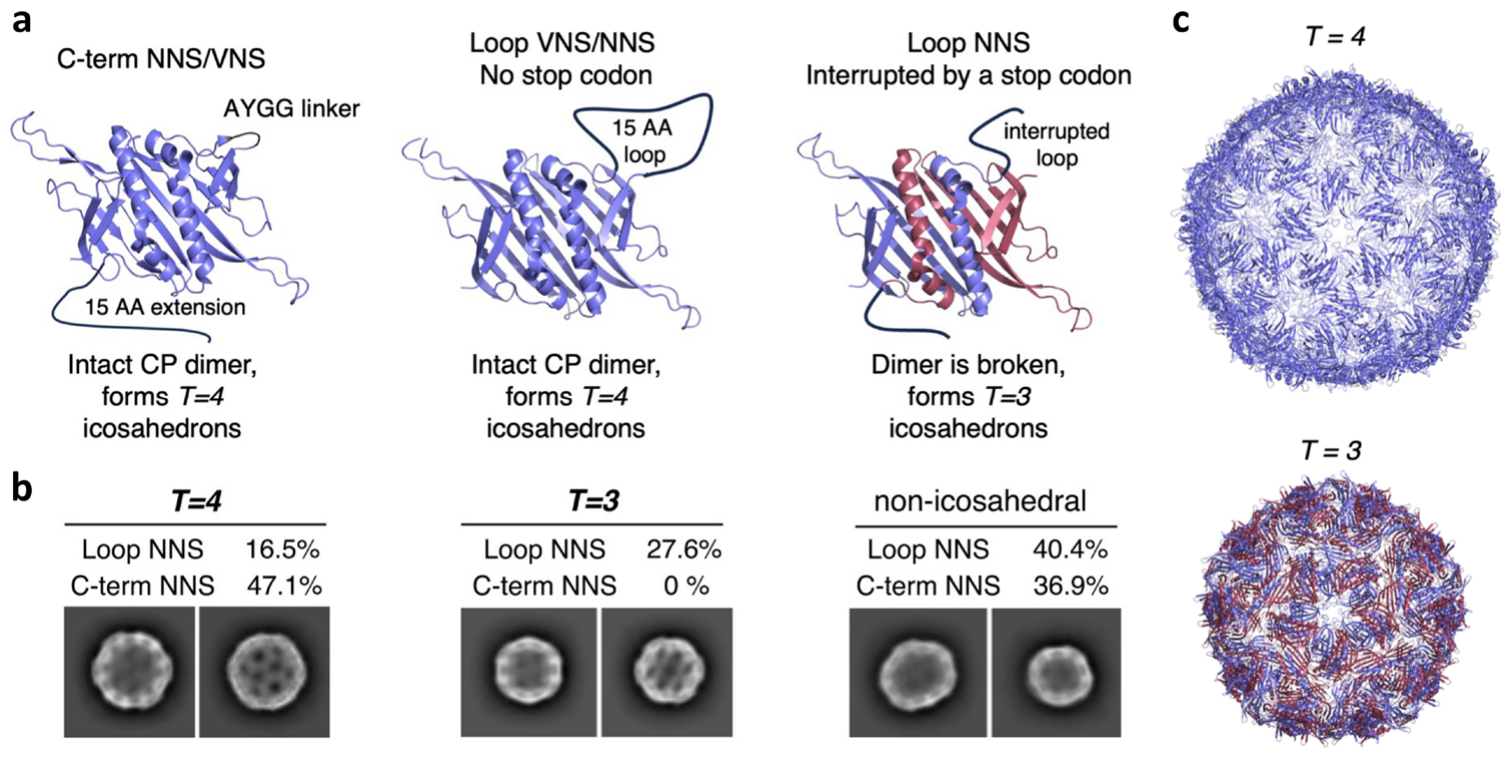
Cryo-EM analysis of the PP7 loop and C-term libraries. (a) PP7 dimer coat proteins with C-term NNS or VNS extension (left), with uninterrupted loop NNS or VNS (middle), or with a stop codon in the loop resulting in two PP7 monomer subunits with C-termini extensions (right). (b) Selected cryo-EM 2D class averages corresponding to *T* = 4, *T* = 3, and non-icosahedral cages. (c) Molecular models of *T* = 4 and *T* = 3 icosahedral cages built from the EM maps obtained after 3D refinement of selected particles.

High-resolution cryo-EM images of NNS particle library samples revealed a variety of particle morphologies (Figures 5 and S17). Among 263,024 particles imaged from the G1 loop NNS library, 16.5% were of a size and shape characteristic of *T* = 4 icosahedra, while 27.7% were assigned to the *T* = 3 class. The remainder of the particles did not exhibit icosahedral symmetry and were excluded from further analysis. *T* = 3 structures are expected from those library members with the TAG stop codon in an added loop, creating monomeric VLPs similar to the WT PP7 capsid (Figure 5a). Analysis of the 3 Å resolution maps of *T* = 4 icosahedra revealed extra densities compared to the canonical PP7 dimer, in the same locations as previously reported for a designed loop insertion (designated PP7-a-loop-PP7) (Figure S17b).^10^ Thus, the junction loop peptides inserted in the library cluster as 20 trileaves at the 3-fold icosahedral axes of symmetry and 60 monoleaves at the 2-fold axes. Analysis of 34,016 particle images from the G1 C-term NNS library revealed 47.1% *T* = 4 icosahedral particles and no *T* = 3 capsids (Figure 5b). The extra densities of the well-ordered *T* = 4 capsids, resolved to 3.2 Å resolution, were found at the same locations as the previously described dimer particle with ZZ-domains added at the C-termini (Figure S17d).^10^ Surprisingly, in both sets of library constructs, we observed several recurring distinct non-icosahedral assemblies, collectively making up 37–40% of the library pool. We speculate that the non-icosahedral assemblies are representing different assembly symmetries, similar to those previously reported for Qβ VLPs.^35^ However, it will take a larger data set than obtained here to discern the detailed nature of these individual assembly states.

### Validation of Assembled VLP Variants from the NNS and VNS Libraries

The appearance of selected sequences in these experiments means only that enough VLPs were produced of each variant to detect by next-generation sequencing. To be of use for most practical applications, such noninfectious particles must be readily generated in substantial quantities. To verify that particles arising from these unbiased selections could fulfill this requirement, we chose 10 sequences of varying frequencies, hydropathy, peptide charge, and cysteine content from each of the four G2 cDNA libraries.

**Figure 6:**
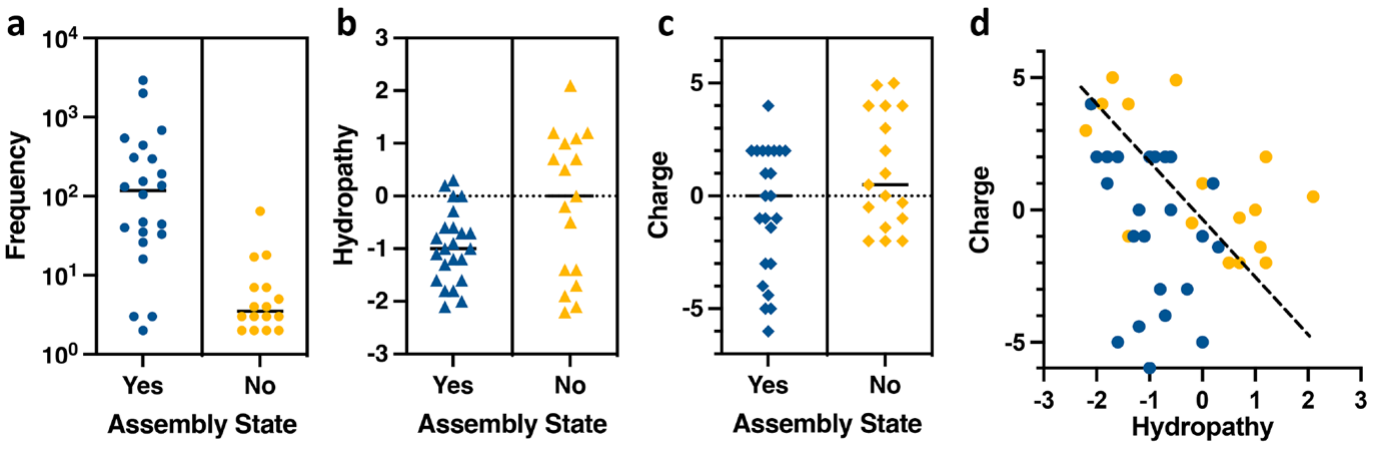
Relationship between the assembly state of PP7 dimer variants selected from the G2 libraries and their frequency in the G2 cDNA library (a), hydrophobicity (b), and charge (c). The solid black lines mark the median (yes = isolated in significant quantities upon individual expression; no = little or no particles isolated upon individual expression). (d) Relationship between hydropathy and charge of the added peptide in successful (blue, significant yield) and unsuccessful (orange, insignificant or no yield) particles. The dashed line is an approximate boundary between a successful and unsuccessful combination of properties.

Forty capsid sequences were individually cloned, expressed, and purified using the same methodology employed for generation and purification of the libraries. “Successful” expression was initially assessed by the appearance of a discernible band at the right location in a sucrose ultracentrifugation gradient at the end of the purification process, indicative of a yield of at least 0.1 mg/L of expression culture. The results, summarized in Figure 6 and Table S2, showed that VLP variants appearing with more than approximately 15 copies in the cDNA library were much more likely to express smoothly, usually yielding at least 4–6 mg of pure particles per liter of culture (Table S2). Thus, the frequency of appearance in the sequenced library is a reasonable indication of “fitness” in the context of this study, showing that VLP assembly and isolability are indeed a challenging function to achieve.

Indeed, the copy number in the selected library was a much more reliable predictor of useful expression yield than either charge or hydropathy of the peptide insertion or addition (with the possible exception of strong negative charge). Successful VLP variants included peptides with GRAVY (grand average of hydropathy) values from +0.3 to −2 (Figure 6b) and calculated charge of +4 to −6, although skewed to the hydrophilic and negatively charged ends of those ranges (Figure 6c). Only two highly abundant library members (loop VNS-6, 65 copies; C-term VNS-4, 47 copies) failed to express individually; these were either unfavorably hydrophobic or positively charged. However, when considered together (Figure 6d), a trade-off is suggested by which more positively charged peptides can be accommodated by a greater degree of hydrophilicity.

## Conclusion

Virus-like particles have proven to be useful in a wide variety of application areas, most notably comprising safe and effective platforms for immunization, imaging, and drug delivery. Their utility relies on their practical advantages: stability, ease of production, and tolerance for surface modifications to display functional molecules. The foundational property of all of these is the ability of VLP capsid proteins to self-assemble during production in the host cell. However, no predictive understanding exists of how a peptide sequence affects self-assembly when such sequences are not engaged in obvious intermolecular interactions in the capsid structure. The great tolerance of natural viruses to mutational variation suggests that capsid assembly can be reliably programmed, but our anecdotal experience with VLPs over many years has taught us that it is not to be taken for granted: even point mutations can give rise to dramatic differences in assembly competence and properties in unpredictable ways.^20,21^ Indeed, even the impressive ability to design self-assembling particles from first-principles^36,37^ does not allow one to reliably program self-assembly of desired structures into mutational variations,^38^ and installing other properties such as genome packaging is best done by including an experimental evolutionary approach.^39^

To add to previous explorations by others of single-point and tripeptide insertions in a related particle,^24−26^ we incorporated randomized 15-mer peptide sequences in the middle and at the end of the PP7 dimer VLP. Although our study focused on sampling representative libraries rather than exhaustively exploring the entire sequence space, it provided two valuable insights. First, negatively charged and hydrophilic peptides encouraged proper self-assembly, and cysteine and bulky amino acids, such as tryptophan, tyrosine, and phenylalanine, disrupted this process. The removal of these four deleterious residues increased the total number of unique PP7 dimer VLPs capable of self-assembling by approximately 2–4 times. Second, selection and enrichment was successful for the desired property of assembly competence, as revealed by the substantial increase in average frequency of selected sequences in the library (Table 1) and the clear dependence of VLP production on that frequency (Figure 6a). This suggests that other functional properties may also be accessed by this method.^40^ In the present case, only one round of selection was needed to arrive at a stable distribution of peptide properties, presumably because the library sizes were small relative to those usually used in directed evolution (but larger than those of VLP libraries reported thus far).

More work will be needed to establish additional design rules for peptide insertions, additions, and mutational changes in virus-like particle structures. For example, we expected obvious differences to emerge in the fitness landscapes for C-terminal extensions vs inserted loops, but the outcome was more subtle. Thus, general trends of hydropathy and charge were similar for these libraries (the general patterns for C-term and loop libraries resemble each other in Figures 4 and S15, panels a vs b and c vs d). However, the loop sequences seemed to be more sensitive to these factors (Figure 3) and the individual patterns of amino acid enrichment vs position were different for loops and extensions (Figures 4, S15). We look forward to further exploration of these and other themes with the aid of coat protein mutational libraries.

## Experimental Section

### Plasmids

Our previously reported PP7 dimer expression plasmid^22^ was modified to accommodate independent double digestion sites with a green fluorescent protein (GFP) gene insertion for the ease of cloning the library. The pET28a vector harboring the PP7 dimer with C-terminal extension was modified with 5′-NheI-sfGFP-MfeI-3′ insertion, while the pCDF1b vector harboring the PP7 dimer with loop insertion was modified with 5′-KpnI-sfGFP-MfeI-3′ insertion. The plasmids used for the loop insertion libraries contained the hairpin loop^29^ encoded after the coding region of coat protein, while it was eliminated from the plasmids used for C-terminal extension libraries.

### Library Generation

Single-stranded DNA oligonucleotides coding for the randomized 15NNS or 15VNS C-terminal and loop insertion libraries were purchased from Eurofins at a 200 nmol scale with high-purity, salt-free (HPSF) purification. All the library oligonucleotides and primers for amplifying the library fragments are listed in the Supporting Information. For the initial library generation, the single-stranded 15NNS or 15VNS DNA oligonucleotides were PCR amplified, and the double-stranded DNA products were purified and used in the subsequent double restriction enzyme digestion. The overnight digested products were purified by 2% agarose gel and subsequently ligated into their respective expression vector using a 1:10 vector/insert molar ratio (4 °C, overnight). The ligated products were transformed into high-efficiency NEB 5-alpha chemically competent *E. coli* cells. A small fraction of the transformed cells was serially diluted and plated for determining the transformation efficiency, while the majority of the transformed cells were used to set up overnight culture for plasmid isolation. The isolated plasmid library was subjected to Sanger sequencing to verify the 15NNS and 15VNS insertion. Details of library expression, VLP purification and characterization, and cryo-EM analysis are provided in the Supporting Information.

### Encapsulated RNA Isolation and Purification

The RNA encapsulated inside the PP7 dimer VLP library was extracted as previously described.^41^ Briefly, the particle library was first treated with 10 μg/mL RNase A at 37 °C for 1 h, followed by addition of TRIzol reagent (Invitrogen) following the user’s guide to inactivate the enzyme and extract VLP-encapsidated RNA. The aqueous layer containing RNA was carefully removed and further purified using the Monarch RNA cleanup kit (NEB). The RNA was eluted in 15 μL of RNase-free water and used immediately for cDNA synthesis or stored at −80 °C.

### cDNA Synthesis and Purification

cDNA was synthesized by the ProtoScript II first-strand cDNA synthesis kit (NEB) using its d(T)23 VN primer. A 5 μL fraction from the cDNA synthesis was used directly as template in a 50 μL PCR to make more 700–800 bp dsDNA amplicons with PP7 coding sequence specific primers. The dsDNA amplicons were purified by a desalting column, and the column elution was used for both downstream digestion and second generation of library subcloning as well as library sequencing material.

### Subcloning and Expression of the Second-Generation Libraries

The dsDNA amplicons were digested by either the NdeI/MfeI pair for the C-terminal extension library or the NdeI/XhoI pair for the loop insertion library to generate 460–470 bp fragments. The digested fragments were ligated into the respective digested vectors (treated with the corresponding restriction enzyme pair). The ligated plasmid library was transformed and verified in the same manner as that for the first generation. The verified second-generation library was then transformed into BL21(*DE3*) cells for VLP library expression, as described above.

### Sample Preparation for High-Throughput Sequencing

The cloned plasmid library, the transformed plasmid library, and synthesized cDNA from extracted RNA were used as templates in two rounds of PCRs to add Illumina sequencing adapters and indexes according to the Illumina 16S Metagenomic sequencing library preparation guide. The final product was purified via BluePippin (Sage), and the quality of the purified product was examined by a DNA bioanalyzer. All of the library samples were combined and sequenced by MiSeq high data output mode in collaboration with the Molecular Evolution Core at the Georgia Institute of Technology.

### NGS Analysis

The raw DNA output from NGS was filtered by an in-house-designed MATLAB code to ensure the constant 5′ and 3′ regions of the amplicons were not mutated or mis-sequenced, and the randomized region followed the intended 15NNS or 15VNS coding sequence. The DNA sequences that passed these filters were translated to the corresponding amino acids, and only those with a frequency of ≥2 were included in downstream analysis to enhance the robustness of the data.

## Supporting Information Available

Experimental details of library expression, VLP purification and analysis, cryo-EM sample preparation, data collection and processing, and additional data (PDF)
