# Supplementary material for: Exploring the Landscape of the PP7 Virus-like Particle for Peptide Display
Source: ACS Nano. 2023 Sep 5;17(18):18470–80. doi: 10.1021/acsnano.3c06178 (PMC10540251; doi:10.1021/acsnano.3c06178)
Supplement: Supplementary file 1 — nn3c06178_si_001.pdf [file nn3c06178_si_001.pdf]

Supporting Information

Exploring the landscape of the PP7 virus-like particle for peptide display

Parisa Keshavarz-Joud,[1#] Liangjun Zhao,[1#] Daija Bobe,[2 §] Carolina Hernandez,[2 §] Mykhailo Kopylov,[2 §] Laura Y. Yen,[2] Naima Djeddar,[3] Brianna Thompson,[1] Caleb Connors,[4] Greg Gibson,[4] Anton Bryksin,[3] M.G. Finn[1,4] *

[1] School of Chemistry and Biochemistry, Georgia Institute of Technology, 901 Atlantic Drive, Atlanta, GA, 30306, USA
[2] New York Structural Biology Center, 89 Convent Ave, New York, NY, USA
[3] Parker H. Petit Institute for Bioengineering and Biosciences, Georgia Institute of Technology
[4] School of Biological Sciences, Georgia Institute of Technology, 901 Atlantic Drive, Atlanta, GA, 30306, USA

[#] Authors contributed equally
[§] Authors contributed equally
* Correspondence: M.G. Finn, e-mail: mgfinn@gatech.edu

Table of Contents

Library expression and VLP purification 2
PP7 particle analysis 2
CryoEM sample preparation, data collection and data processing 3
Supplementary Figures 4
  Figure S1 4
  Figure S2 5
  Figure S3 6
  Figure S4 7
  Figure S5 8
  Figure S6 8
  Figure S7 9
  Figure S8 9
  Figure S9 10
  Figure S10 11
  Figure S11 12
  Figure S12 13
  Figure S13 14
  Figure S14 15
  Figure S15 16
  Figure S16 17
  Figure S17 18
Supplementary Tables 19
  Table S1 19
  Table S2 20
  Table S3 21
  Table S4 22
  Table S5 23
  Table S6 24
  Table S7 25
  Table S8 26
  Table S9 27
  Table S10 28
References 28

Library expression and VLP purification

50 μL of *E. cloni* EXPRESS BL21*(DE3)* electrocompetent cells (Biosearch Technologies) were transformed with 400 ng of plasmid library by electroporation. After transformation, 10 μL of the transformed cell suspension was serial diluted for plating and determining transformation efficiency, while the rest was used for setting up overnight culture, of which 5–10 mL was sampled the next morning for isolation of the transformed plasmid library. The remainder of the overnight culture was used to inoculate at 1:50 ratio into fresh 2YT media supplemented with appropriate antibiotics. The culture was incubated at 37 °C with shaking (250 rpm) until an OD_{600} value of 0.8–1.0 was reached. VLP protein expression was induced by adding IPTG to a final concentration of 1 mM and the expression culture was kept at room temperature with shaking (250 rpm) overnight. Cells were harvested by centrifugation at 6,000 rpm, and the cell pellets were processed immediately or stored at -80 °C. The cell pellet from 500 mL culture was resuspended in 80–100 mL potassium phosphate buffer (0.1 M, pH 7.0) and sonicated at 60–65% power for 15 min with 5-second bursts separated by 5-second intervals. The centrifugation cleared lysate was precipitated with 0.265 g/mL ammonium sulfate at 4 °C for about 3 hours. The crude protein library precipitate was collected by centrifugation at 13,000 rpm for 10 minutes, and the protein pellet was suspended with potassium phosphate buffer (0.1 M, pH 7.0) and kept at 4 °C with mild shaking to allow complete dissolution. The lipids and membrane proteins in the crude library sample were removed by 1:1 n-butanol:chloroform organic extraction. After recovering the top aqueous layer containing VLP library, sucrose (10–40% w:v). density gradient was used to further polish the library sample. The PP7 VLP library fractions was collected and pelleted out by ultracentrifugation at 68,000 RPM (Beckman Type 70 Ti Rotor) for 2 hours. The library protein pellet was dissolved in the potassium phosphate buffer (0.1 M, pH 7.0) and filtered through 0.22 μm filter.

PP7 particle analysis

PP7 protein concentration was measured using the Coomassie Plus Protein Reagent (Pierce) with bovine serum albumin as standard. Molecular weight distributions of assembled PP7 VLP libraries were assessed by LC-MS (ESI-ToF, Agilent), although we do not assume that all library members are detectable by this technique. Accurate mass measurements on individual VLP sequences were determined on the same instrument. For all mass spectrometry analyses, VLP samples were first denatured by incubation of 10 μL of 0.5 mg/mL VLP solution with a mixture of 2.5 μL of 1M DTT and 2.5 μL of 1M urea for 5 minutes at room temperature) followed by dilution (90 μL water) and centrifugation (13,000 rpm for 2 minutes) to remove aggregates. Each sample (5 μL, corresponding to 250 ng VLP) was introduced to the mass analyzer after passage through a C3 reversed-phase HPLC column (0.5 mL/min; step elution profile = 98/2 H_2O/MeCN for 2.5 min; 20/80 H_2O/MeCN for 4.5 min; column wash with 100% MeCN, all elution solvents containing 0.1% formic acid).

Particle sizes were assessed by DLS, TEM, and SEC. All libraries were diluted to 0.1 mg/mL concentration and assessed with a Dynapro DLS plate reader (Wyatt Technologies) in a 384-well plate. TEM samples were prepared by diluting each of the VLP libraries to 0.05–0.1 mg/mL with sterile water. Grids were prepared by applying 6 μL of diluted VLP sample to the carbon surface of 300 mesh lacey formvar/carbon TEM grids (Ted Pella Inc.) and incubating for 90 seconds.

Grids were blotted against a Kimwipe and excess salts were removed by laying the carbon surface of the grid on 1.0 mL water drops for 5–10 seconds and repeating once more. The grids were blotted against a Kimwipe again and negative staining was performed by applying 5 μL of a 2% w/v uranyl acetate stain solution onto the grid for 45 seconds. The grids were blotted and air dried for 5–10 minutes at room temperature. TEM images were acquired with Hitachi HT-7700 operating at 120 kV. Size-exclusion chromatography was performed on a Superose-6 column (0.4 mL/min flow rate with 0.1 M potassium phosphate buffer, pH 7.0). The protein content of library samples was determined by dilution to 1.0 mg/mL and analysis with a Bioanalyzer 2100 Protein 80 microfluidics chip (Agilent) for the protein content.

CryoEM sample preparation, data collection and data processing

R1.2/1.3 quantifoil (carbon foil) holey grids (EMS, 300 mesh) were plasma-cleaned using Solarus Gatan Plasma System in an oxygen-argon environment at 15 W power (30 s for C-term NNS samples; 7 s for loop NNS samples). VLPs were diluted to 1 mg/mL, and an aliquot of 3 µL of sample was applied to the foil side of the grid. Grids were plunge-frozen into liquid ethane on a Leica EM GP automatic plunge freezer (Leica Microsystems) after blotting (1 s for C-term NNS; 3 s for loop NNS). (The indicated differences in these procedural parameters derived from the routine procedures of different users rather than from differences in sample properties.) Frozen grids were clipped in AutoGrid rings and stored in liquid nitrogen until data collection.

C-term NNS data were acquired at the New York Structural Biology Center (NYSBC) on a Krios 1 instrument (dataset 19nov18k) with the following parameters: Gatan K2 camera in counting mode, 70 e^-/A^2 total dose on a specimen level, 10 s exposure fractionated into 50 frames, 1-2 μm nominal defocus range and 1.073 Å calibrated pixel size. A total of 2631 movies were acquired using Leignon [1] for automatic data acquisition. Loop NNS data were acquired at NYSBC on a Krios 2 instrument equipped with Gatan Bioquantum energy filter (dataset 21feb14a) with the following parameters: Gatan K3 camera in counting mode (1x binning), 52 $e^-/Å^2$ total dose, 2 s exposure fractionated into 50 frames, energy filter slit width 15 eV, 1–2 μm nominal defocus and 1.076 Å calibrated pixel size. A total of 1026 movies were acquired using Leginon.

Data processing for both datasets was done using the CryoSPARC 4.2.1 software suite. Movies were first motion-corrected using Patch Motion Correction and CTF was estimated using Patch CTF. Initial particle picks were done using Blob Picker with a set diameter range of 265–320 Å. Extracted particles were ran through 2D classification and generated 2D class averages were used as templates for Template Picker. Particles were extracted with the box size of 420 pixels and classified in 2D. Resulting 2D class averages were inspected visually to select particle subsets corresponding to T=3 and T=4 icosahedral assembly states. Selected subsets were used to generate *ab-inito* model and refined with icosahedral symmetry applied to produce final maps. Maps were analyzed with UCSF Chimera 1.13.[2] To generate the low-resolution maps of surface densities, the T=4 loop NNS and C-term NNS maps were first low-pass filtered to 15 Å resolution and then a 15 Å low-pass filtered map of WT PP7-AYGG-PP7 T=4 (EMD-0344) was subtracted from both.

Supplementary Figures

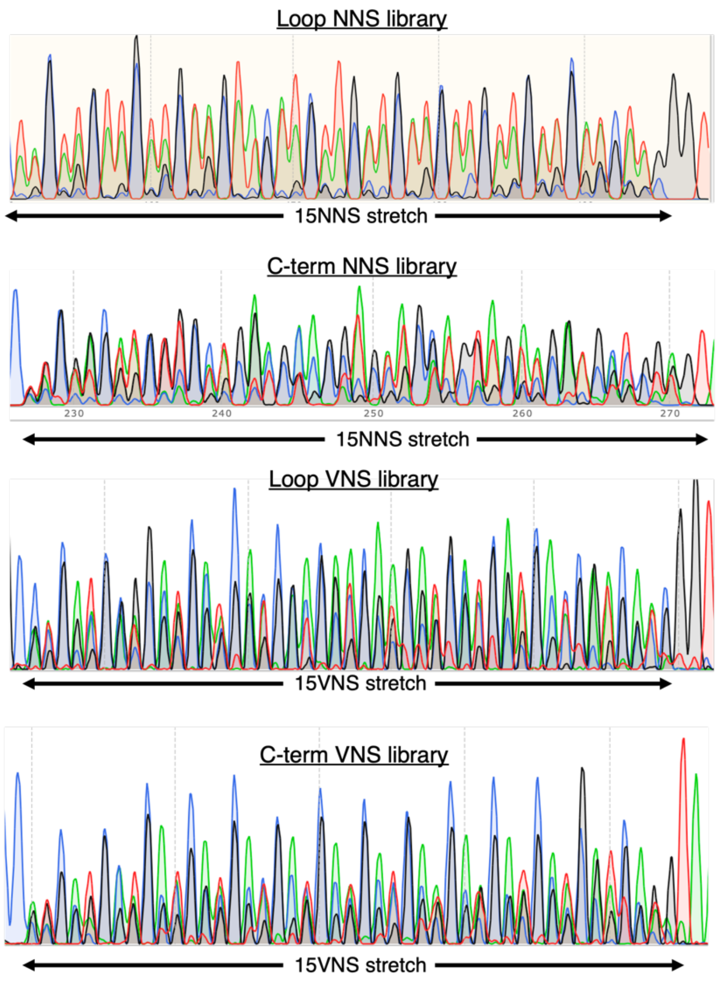

Figure S1. Sanger sequencing traces of the cloned plasmid libraries, showing the success in cloning of the 15NNS and 15VNS stretch in the loop or C-term of PP7 dimer coat protein. A = green, T = red, C = blue, G = black.

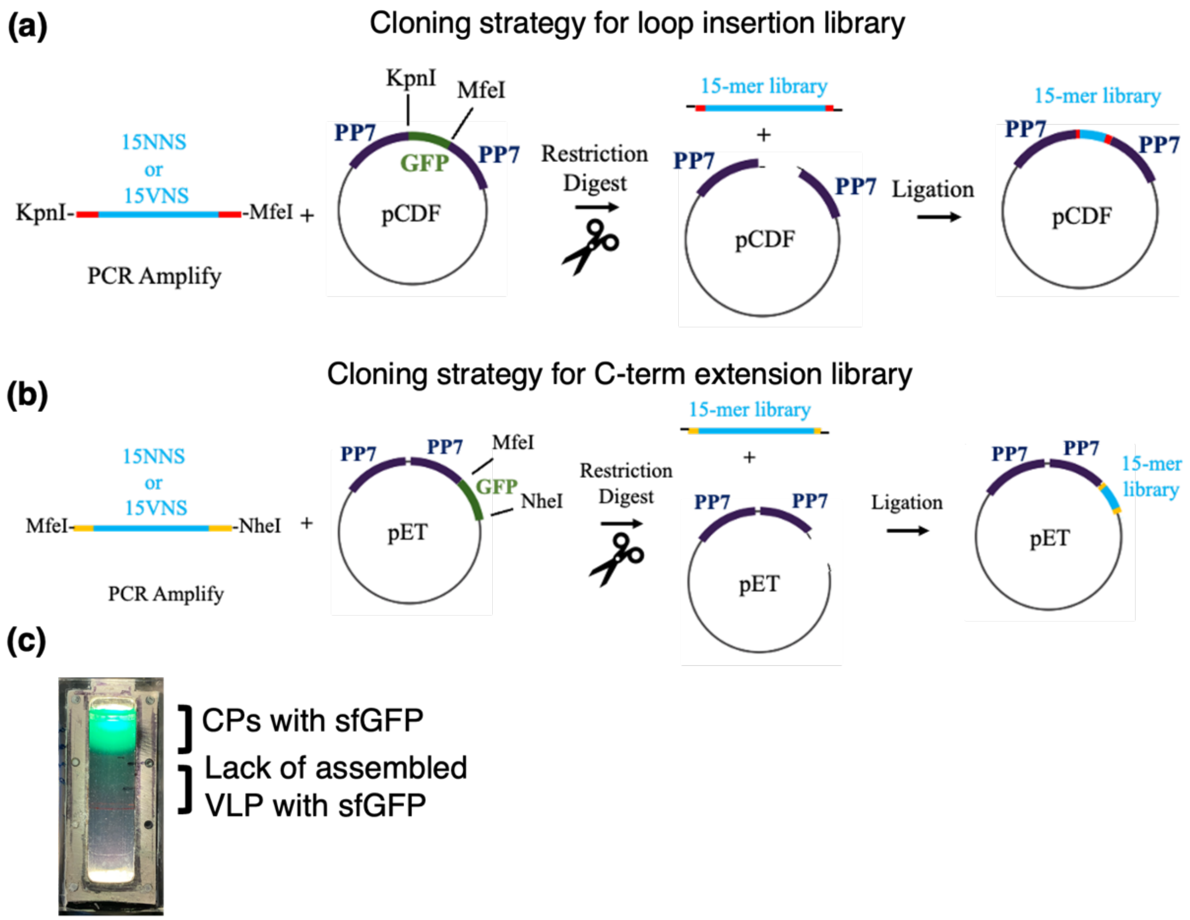

Figure S2. Library cloning strategy. (a, b) Plasmids encoding sfGFP are used as the initial vector since coat protein (CP) variants containing this motif are incapable of assembly into VLPs, as shown by the sucrose gradient (c). This strategy eliminates the interference of any small trace amount of plasmid template carryover in the downstream VLP library analysis.

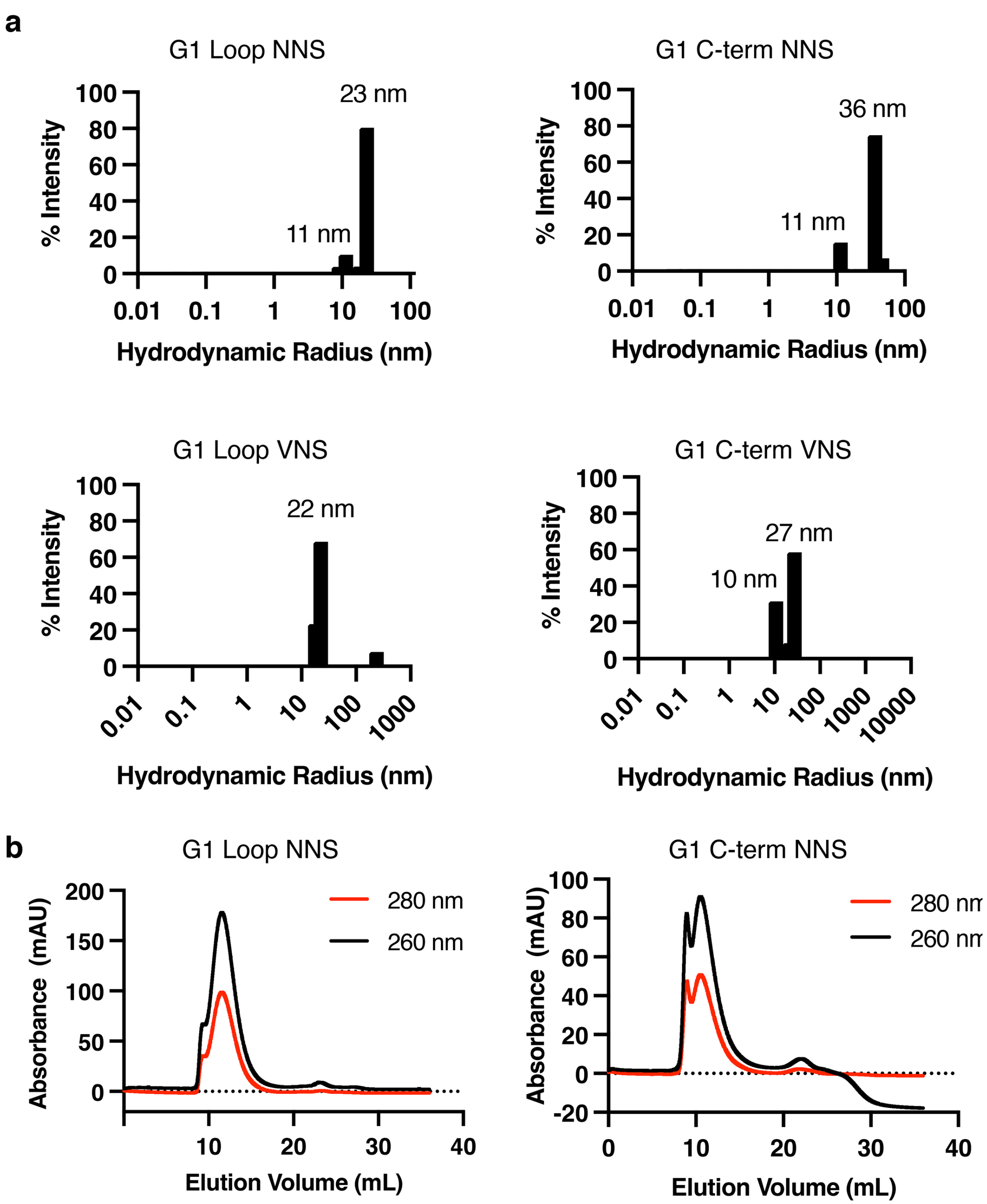

Figure S3. Characterization of particles isolated from generation 1 (G1) NNS libraries. **(a)** Hydrodynamic radii of VLP libraries assessed by dynamic light scattering (DLS). **(b)** Size-exclusion chromatography (SEC) elution profile of VLP libraries with the 260 nm wavelength monitoring the encapsulated nucleic material that co-migrates with the assembled protein particles, monitored by 280 nm signal.

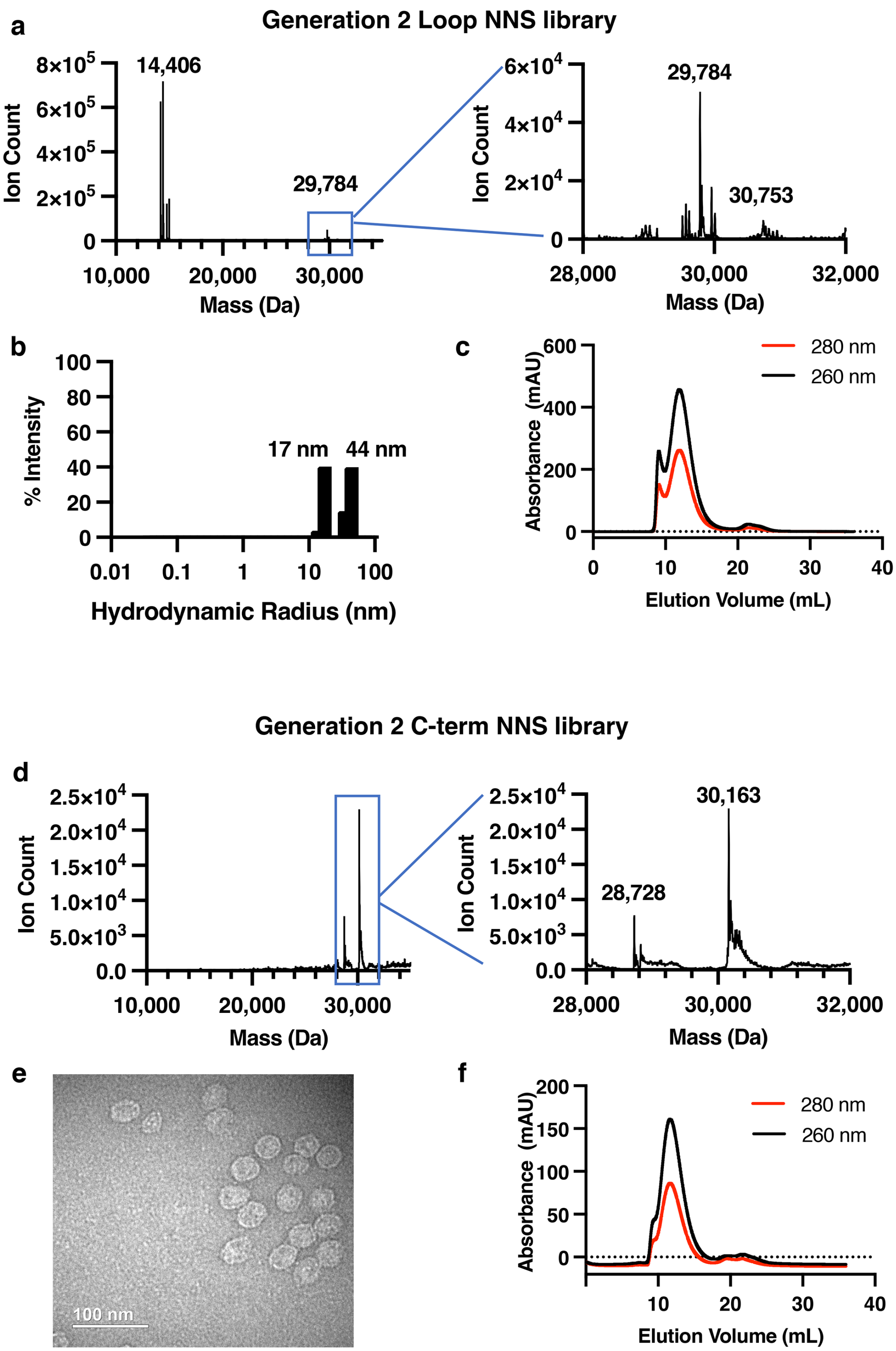

Figure S4. Characterization of particles isolated from generation 2 (G2) NNS libraries. Panels a-c describe the G2 loop library; panels d-f describe the G2 C-term library. **(a,d)** CP mass spectrometry trace of VLP libraries. **(b)** hydrodynamic radius determined by DLS. **(c,f)** SEC profile and **(e)** TEM image of assembled VLPs.

Figure S5. Characterization of particles isolated from generation 2 (G2) VNS libraries. Panels a-b describe the G2 loop library; panels c-d describe the G2 C-term extension library. **(a,c)** hydrodynamic radius determined by DLS. **(b,d)** SEC profile of assembled VLP libraries.

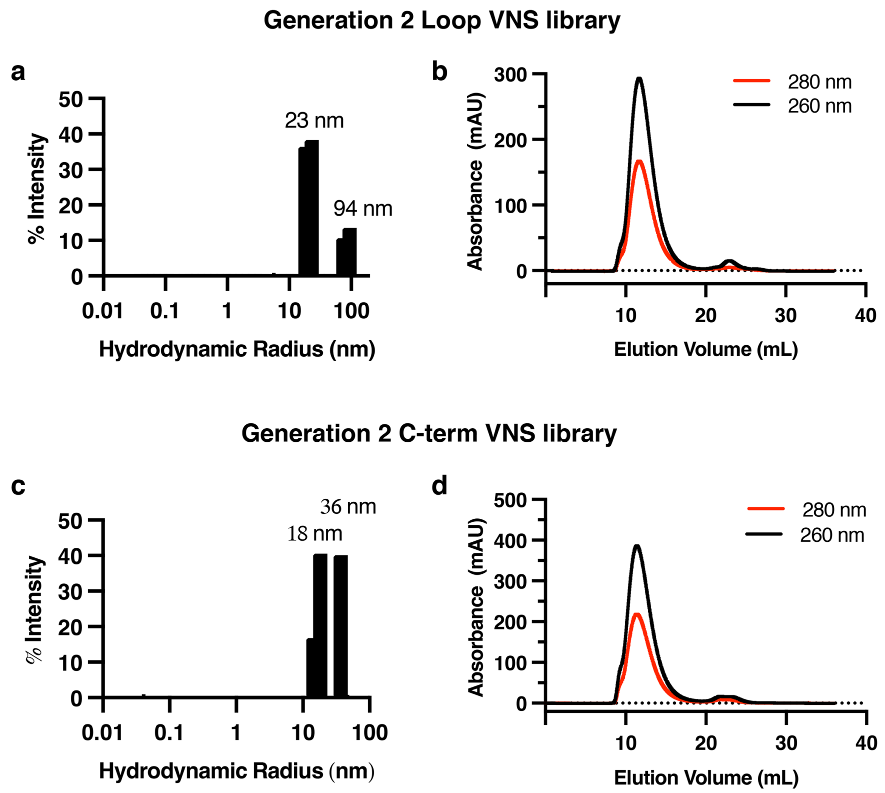

Figure S6. Length distribution of peptides encoded within the G1 loop (left) and C-term (right) NNS transformed libraries.

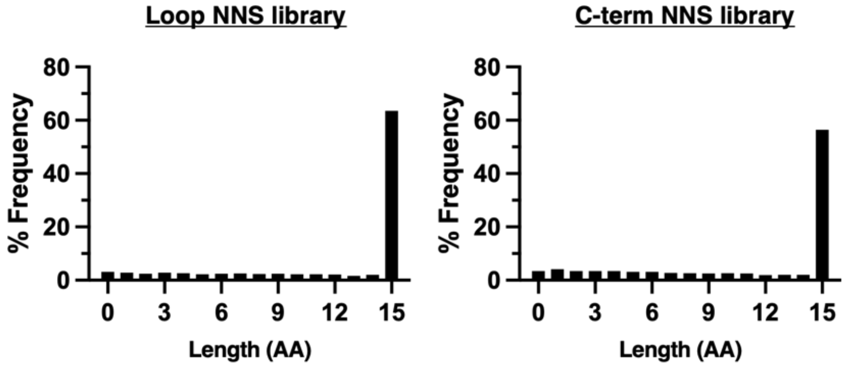

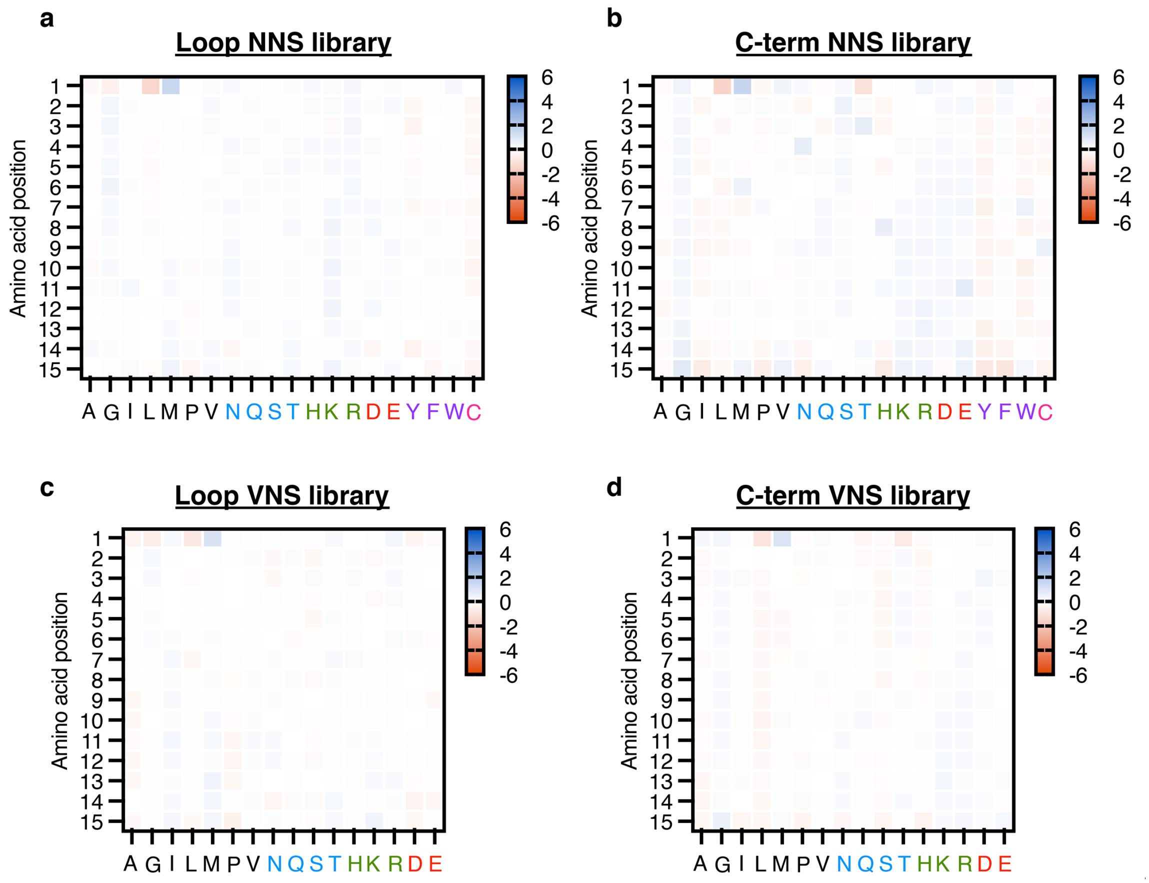

Figure S7. Assessment of bias in the G1 transformed plasmid libraries compared to theoretical codon frequency in the respective NNS or VNS codons. Heat maps represent log_2 of the abundance ratio (cDNA library / transformed library), of each amino acid at each position. **(a)** G1 loop NNS, **(b)** G1 C-term NNS, **(c)** G1 loop VNS, and **(d)** G1 C-term VNS libraries.

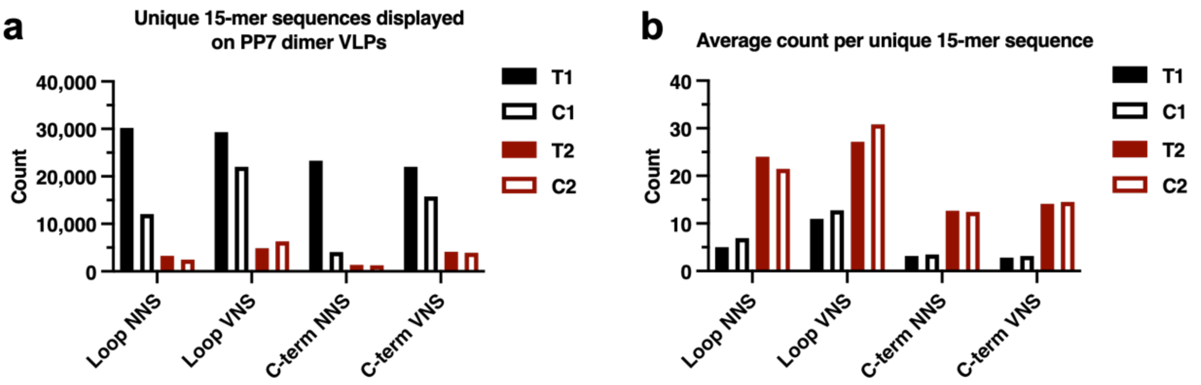

Figure S8. **(a)** Total number of unique 15-mer sequences with frequency ≥ 2 successfully displayed on the PP7 dimer VLPs. **(b)** Average frequency of unique 15-mer sequences successfully displayed on the PP7 dimer VLPs. T1 = generation 1 transformation library (possible sequences expressed in *E. coli*), C1 = generation 1 cDNA library (particles recovered and sequenced), T2 and C2 = transformation and cDNA libraries for generation 2, respectively.

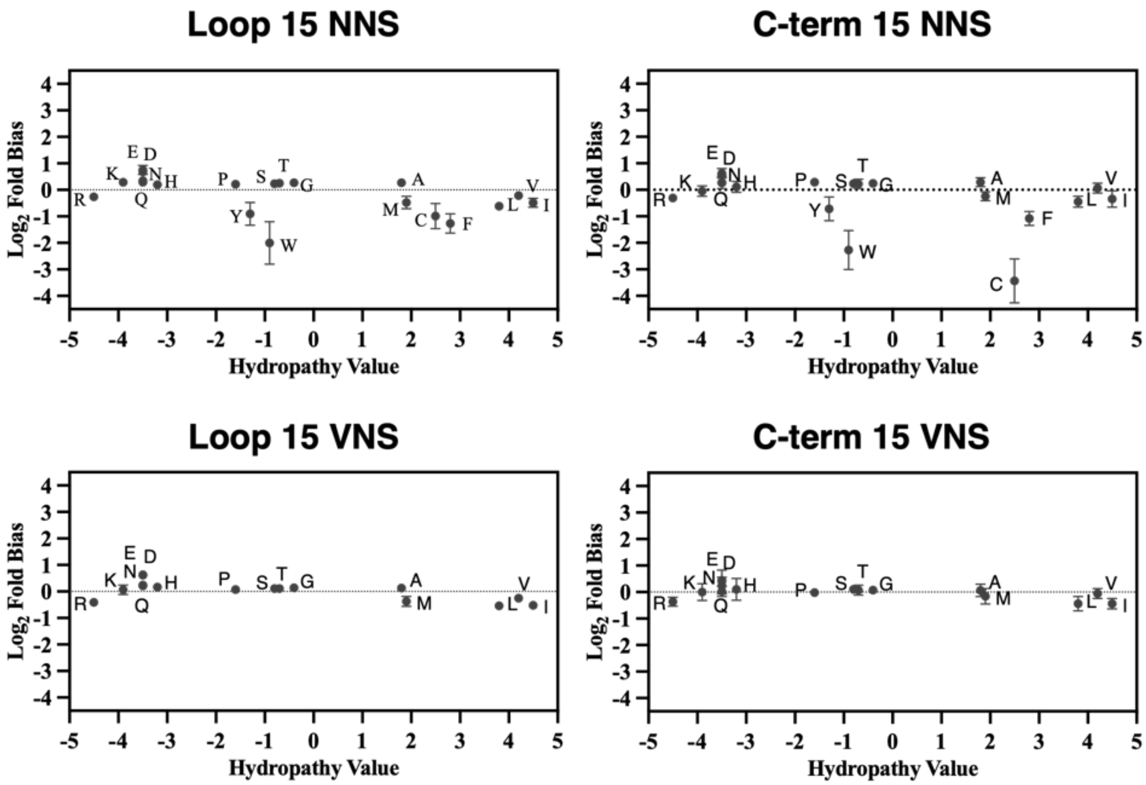

Figure S9. Favorability of amino acids within the G1 cDNA libraries in relation to hydrophobicity. The y-axis values represent average $\log_2$ of the abundance ratio (cDNA library / transformed library) of a given amino acid across positions 1-15 of the extension or loop insert; the error bars represent the global standard deviation for each amino acid that appears within one of the variable peptide segments.

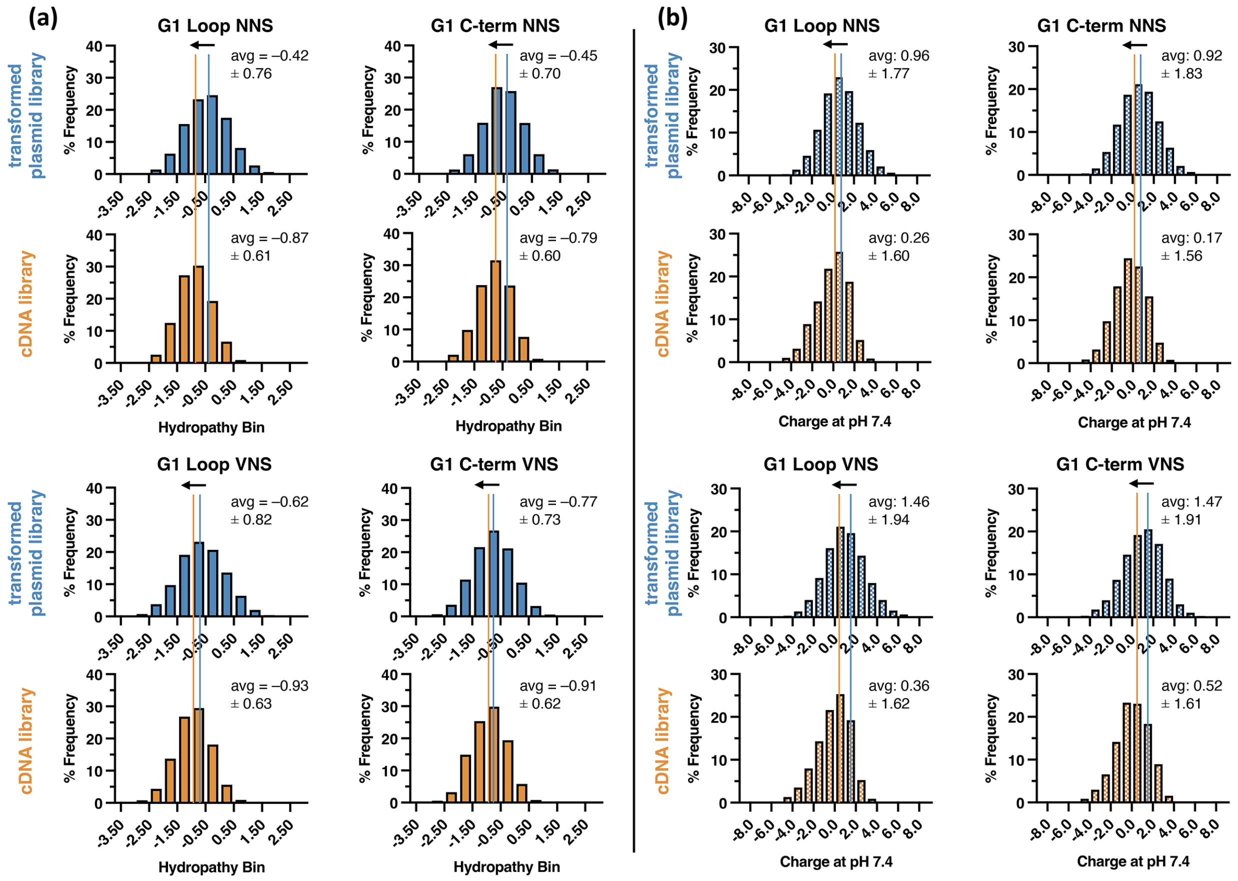

Figure S10. Calculated properties of peptides in the loops or C-termini of the indicated first-generation PP7 dimer VLP libraries, comparing all of the sequences available for expression (transformed plasmid library) to those of the assembled, isolated, and sequenced particles (cDNA library). **(a)** Hydropathy evaluated by GRAVY score distribution. **(b)** Charge at pH 7.4.

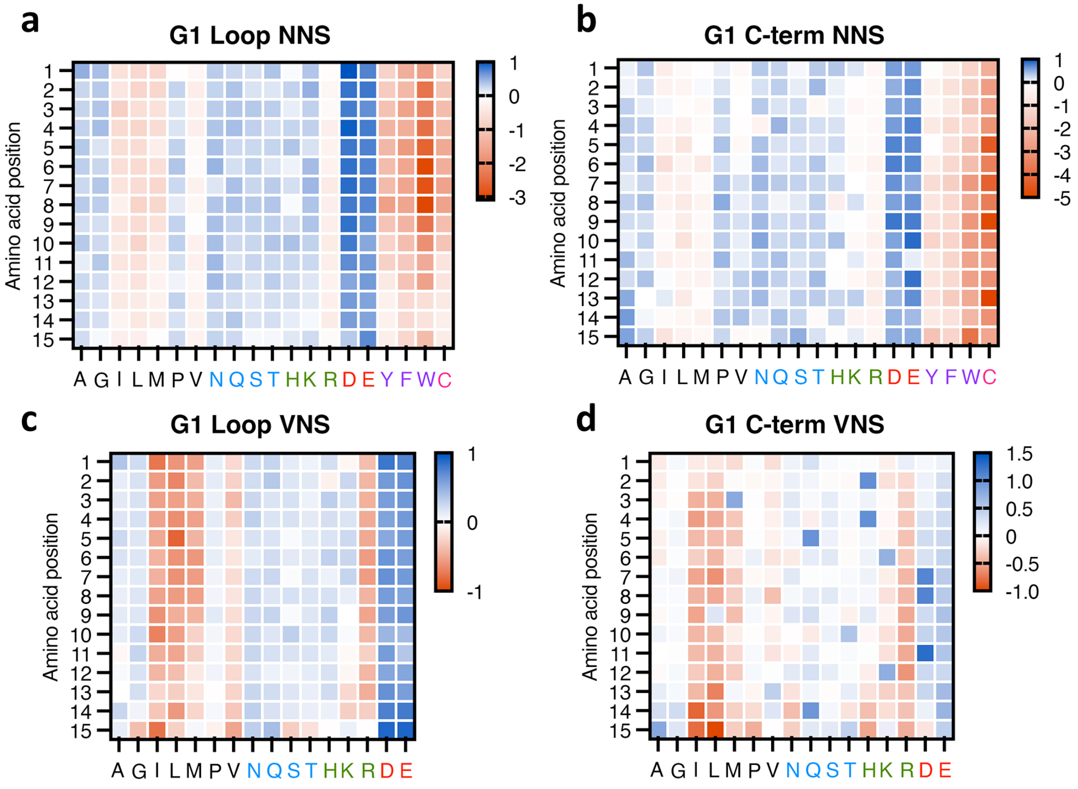

Figure S11. Favorability, measured by $\log_2$ of the abundance ratio (cDNA library / transformed library), re-plot of Figure 4 with the color scale matching the full range of data within each panel. **(a)** Generation 1 (G1) loop NNS, **(b)** G1 C-term NNS, **(c)** G1 loop VNS, **(d)** G1 C-term VNS. Amino acids are grouped by their properties indicated by the color of the single-letter code (hydrophobic = black, polar/uncharged = blue, basic and positively charged = green, acidic and negatively charged = red, aromatic = purple, and thiol containing = pink).

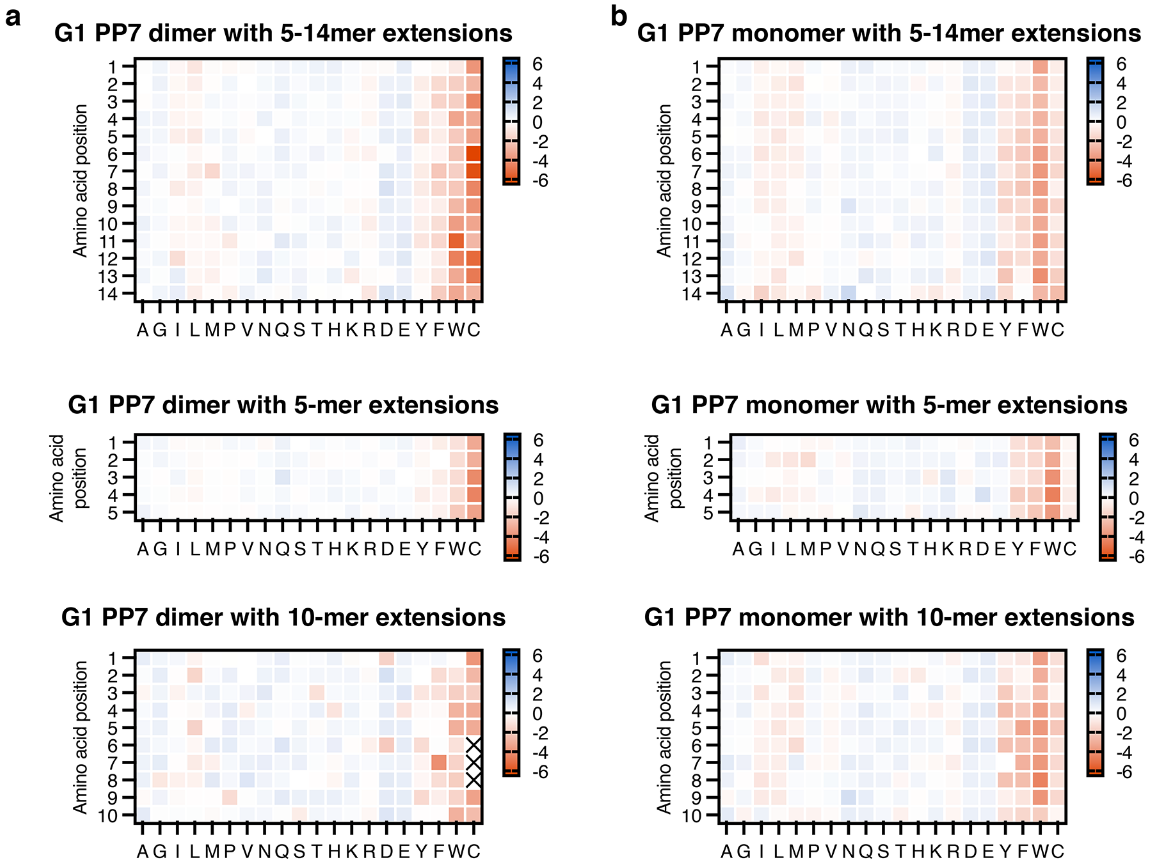

c

Extension Length on PP7 dimer	# transform. sequence	# unique transform. sequence	# cDNA sequence	# unique cDNA sequence
5	5,507	1,522	6,000	1,345
6	5,452	1,525	5,087	1,124
7	4,663	1,350	3,658	862
8	4,441	1,280	3,123	731
9	4,039	1,165	2,261	599
10	4,575	1,178	1,741	452
11	4,325	1,087	1,480	358
12	3,072	906	941	249
13	3,221	988	827	216
14	2,976	917	663	182

d

Extension Length on PP7 monomer	# transform. sequence	# unique transform. sequence	# cDNA sequence	# unique cDNA sequence
5	6,750	1,068	18,625	1,062
6	7,469	1,172	17,990	1,028
7	7,522	1,194	17,527	1,014
8	7,014	1,138	14,240	850
9	7,009	1,163	13,342	791
10	6,465	1,060	10,063	647
11	6,513	1,096	10,307	620
12	5,795	997	8,474	531
13	4,349	841	5,051	400
14	5,292	1,057	4,734	417

Figure S12. Favorability (a, b) and count (c, d) of truncated peptide extensions appended to PP7 dimer and PP7 monomer capsids, as the result of random TAG stop codon appearance in the G1 C-term NNS and loop NNS libraries, respectively. Favorability is measured by $\log_2$ of the abundance ratio (cDNA library / transformed library) of each amino acid at each position. The favorability heat plots represent the combined results for 5- to 14-mers (a, b; top panels), exclusively 5-mers (a, b; middle panels), and exclusively 10-mers (a, b; bottom panels). The latter two are included to show that specifically-sized extensions give similar results as the pooled analyses. Crossed out cells reflect the omission of the indicated amino acid at that position in the cDNA library. (c, d) The number of total and unique peptide sequences (appearing at least twice) of particles isolated from each library.

a

Repli-cate	Oligo library source	Illumina	Experi-menter	yield (mg)	# transform. sequences [a]	# unique transform. sequences [a]	# cDNA sequences [b]	# unique cDNA sequences [b]
**1**	Eurofins	MiSeq	LZ	0.6	74,461	23,336	14,145	4,064
**2**	IDT	MiSeq	PK	0.25	32,217	9352	26,939	2,440
**3**	IDT	MiniSeq	LZ	0.5	430,082	32,437	228,629	5,411

a) from transformed *E. coli* cells (transformation libraries). b) from isolated particles (cDNA libraries)

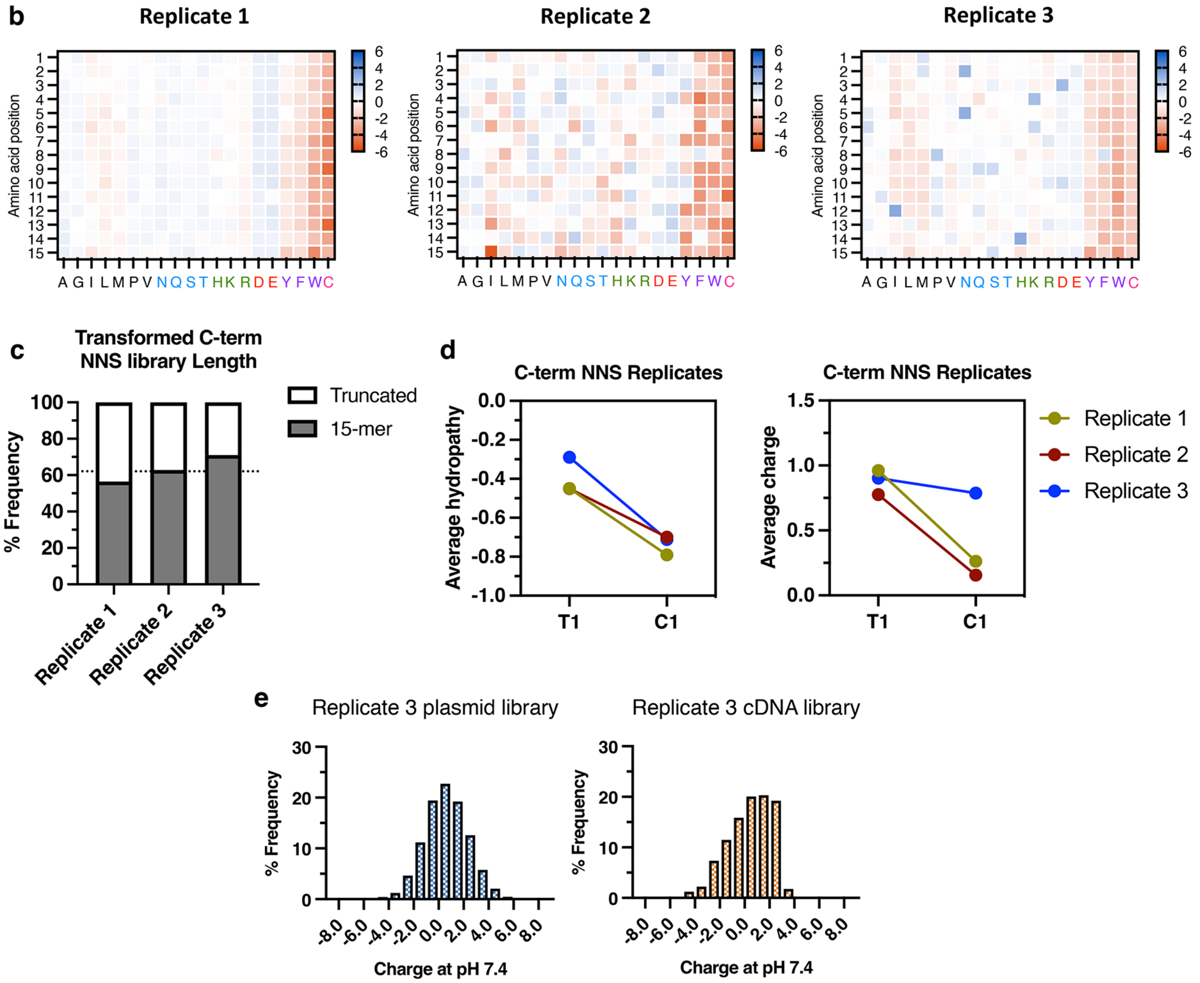

Figure S13. Three of G1 C-term NNS library compared side-by-side. Library replicate 1 was constructed from nucleotide library purchased from Eurofins, whereas nucleotide libraries in replicates 2 and 3 were purchased from IDT. Replicates 1 and 2 were sequenced by Illumina MiSeq, while replicate 3 was sequenced by Illumina MiniSeq. **(a)** Assembled particle yields (from 0.5 L of E. coli culture) and the number of total and unique sequences (appearing at least twice) of particles isolated from each library. **(b)** Heat map showing the log_2 fold bias of each amino acid at various positions of the 15-mer C-terminal extension. **(c)** The length of full 15-mers vs the truncated peptides sequenced in each replicate. **(d)** Change in the hydropathy (left) and charge (right) from the G1 transformed DNA library (T1) and the cDNA generated from assembled VLP variants (C1). (**e**) Calculated properties of peptides in replicate 3 of the plasmid and cDNA libraries in this experiment, showing hydropathy evaluated by GRAVY score (*left*) and calculated peptide charge at pH 7.4 (*right*).

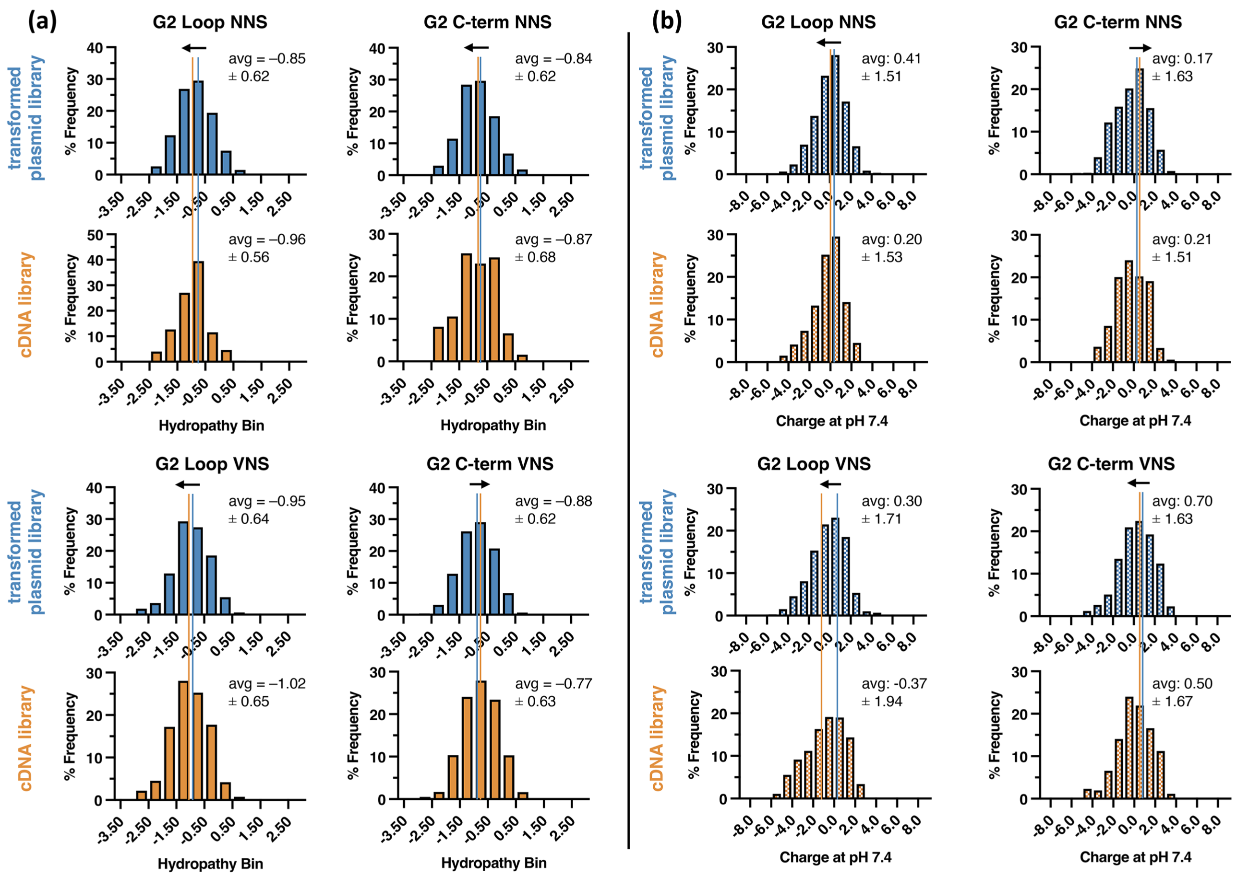

Figure S14. Calculated properties of peptides in the loops or C-termini of the indicated second-generation PP7 dimer VLP libraries, comparing all of the sequences available for expression (transformed plasmid library) to those of the assembled, isolated, and sequenced particles (cDNA library). **(a)** Hydropathy evaluated by GRAVY score distribution. **(b)** Charge at pH 7.4.

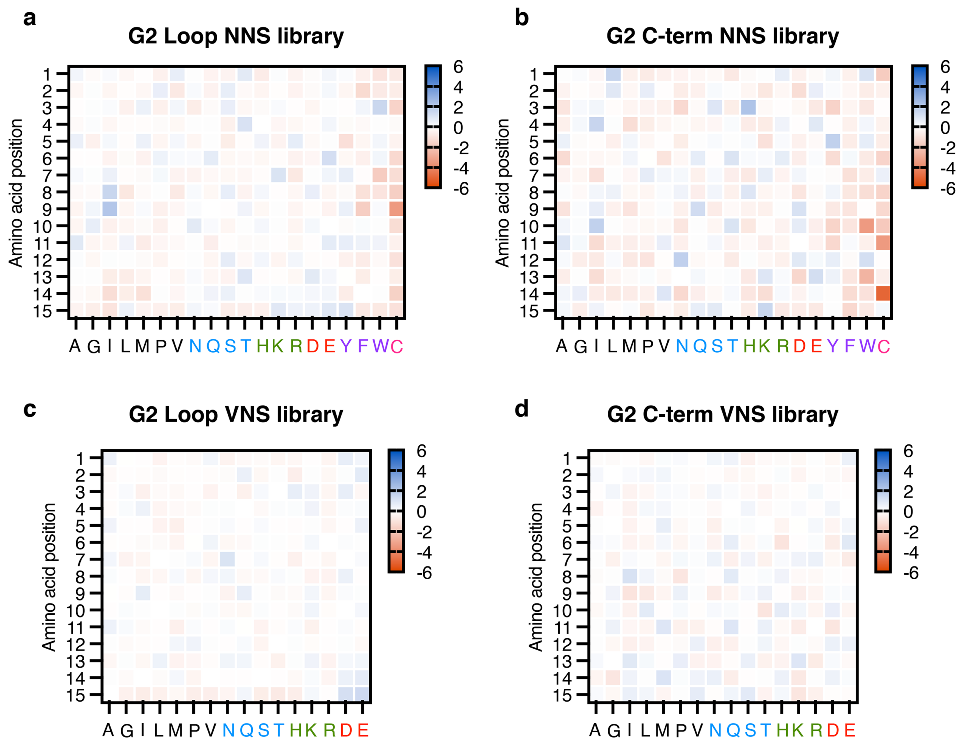

Figure S15. Favorability measured by $\log_2$ of the abundance ratio (cDNA library / transformed library), of each amino acid at each position. **(a)** Generation 2 (G2) loop NNS, **(b)** G2 C-term NNS, **(c)** G2 loop VNS, **(d)** G2 C-term VNS.

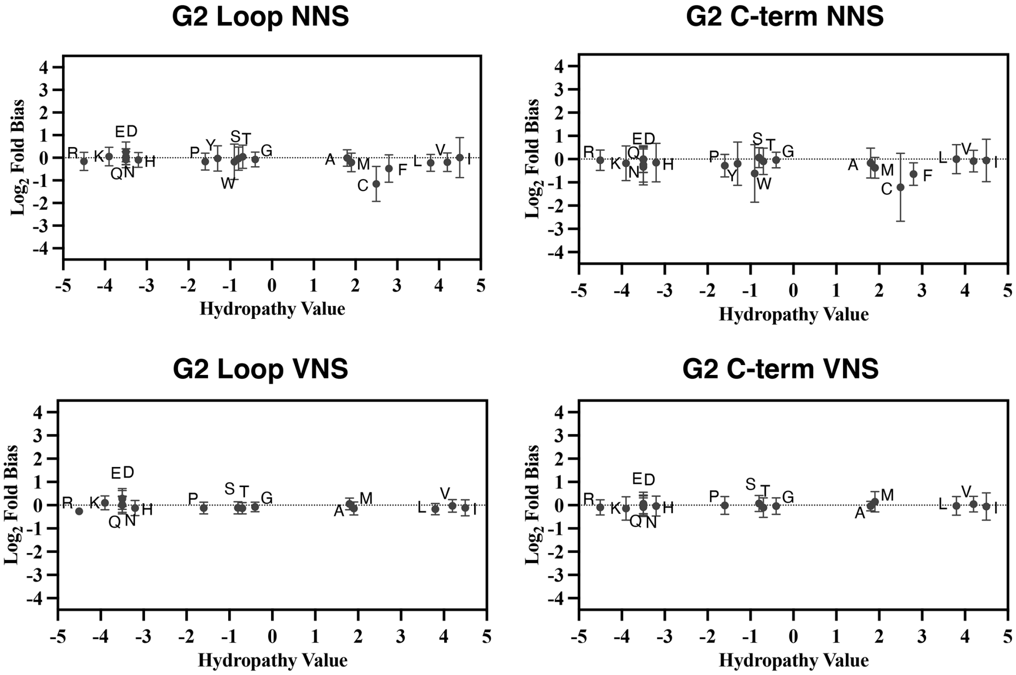

Figure S16. Favorability of amino acids within the G2 cDNA libraries in relation to hydrophobicity. The y-axis values represent average $\log_2$ of the abundance ratio (cDNA library / transformed library) of a given amino acid across positions 1-15 of the extension or loop insert; the error bars represent the standard deviation within these positions.

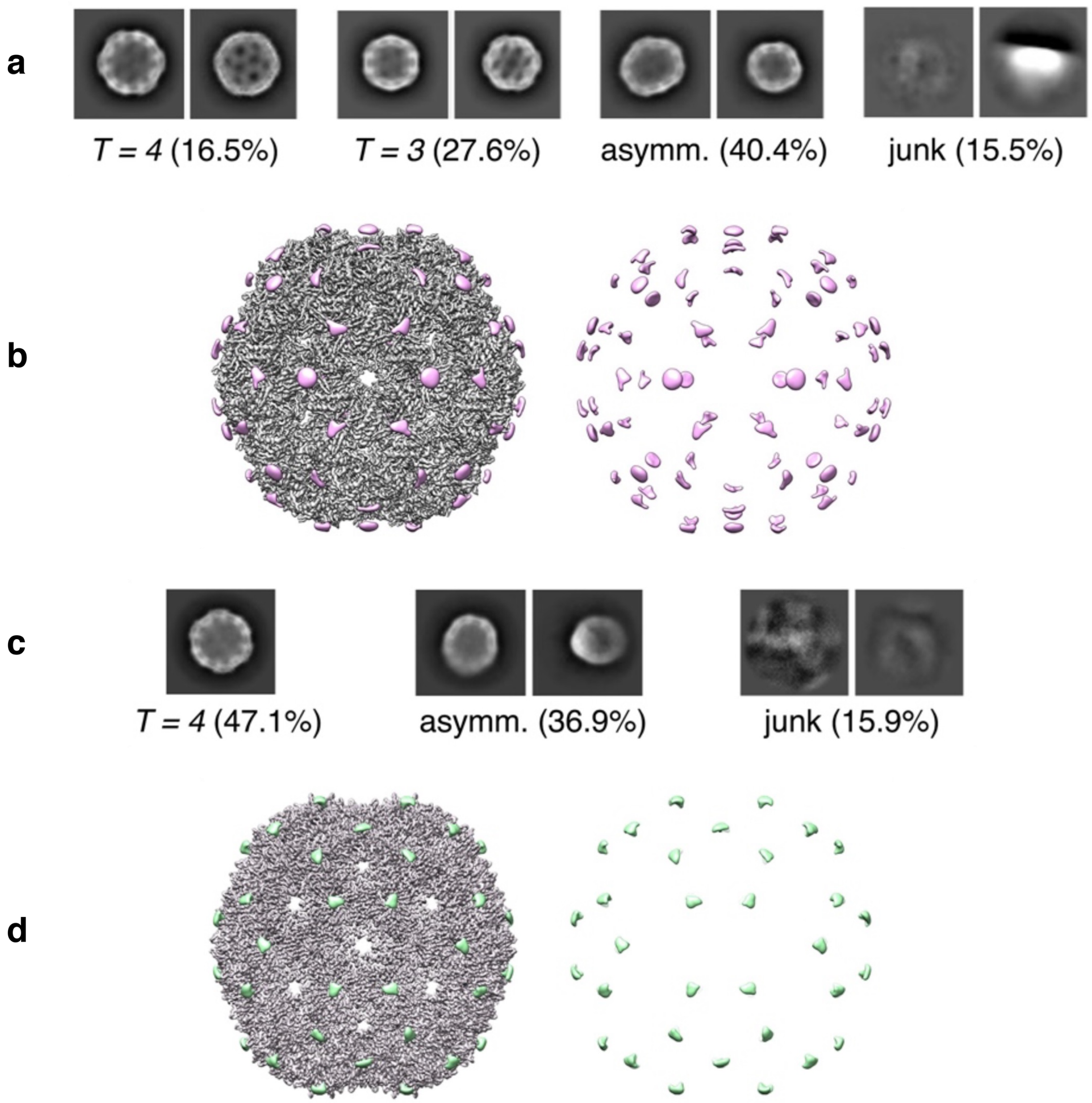

Figure S17. Cryogenic electron microscopy analysis of particle libraries displaying randomized 15NNS sequences. (a) Analysis of 263,024 particles from G1 loop NNS library, identifying icosahedral particles with both *T=3* and *T=4* symmetries. (b) *T=4* class map of G1 loop NNS library (left) with the surface density of the displayed randomized loop peptide highlighted (right). (c) Analysis of 34,016 particles in the G1 C-term NNS library containing well-ordered *T=4* icosahedral symmetry. (d) *T=4* class map of G1 C-term NNS library (left) with the surface density of the displayed randomized peptide highlighted (right). "Junk" refers to protein aggregates with no discernable structure.

Supplementary Tables

Table S1. Sequence of oligonucleotides used for cloning, amplification, and NGS preparation.

Construct	DNA Sequence
Loop NNS oligonucleotide library (KpnI/15NNS/MfeI)	AATCTGGTACCACTTGGTCGCGCATATGGCNNSNNSNNSNNSNNSNN SNNSNNSNNSNNSNNSNNSNNSNNSNNSGGTTCGAAAACAATTGTCCT GTCC
Loop VNS oligonucleotide library (KpnI/15VNS/MfeI)	AATCTGGTACCACTTGGTCGCGCATATGGCVNSVNSVNSVNSVNSVNS VNSVNSVNSVNSVNSVNSVNSVNSVNSGGTTCGAAAACAATTGTCCTG TCC
Loop NNS and VNS oligonucleotide forward primer	AATCTGGTACCACTTGGTCGCGCATATGGC
Loop NNS and VNS oligonucleotide reverse primer	GGACAGGACAATTGTTTTCGAACC
C-term NNS oligonucleotide library (NheI/15NNS/MfeI)	GGTGGCGCTAGCGAAAGCGGCGCCNNSNNSNNSNNSNNSNNSNNSN NSNNSNNSNNSNNSNNSNNSNNSTAACAATTGCGAGCACCACCAC
C-term VNS oligonucleotide library (NheI/15VNS/MfeI)	GGTGGCGCTAGCGAAAGCGGCGCCVNSVNSVNSVNSVNSVNSVNSVN SVNSVNSVNSVNSVNSVNSVNSTAACAATTGCGAGCACCACCAC
C-term NNS and VNS oligonucleotide forward primer	GGTGGCGCTAGCGAAAGCGGCGCC
C-term NNS and VNS oligonucleotide reverse primer	GTGGTGGTGCTCGCAATTGTTA
cDNA amplification forward primer	GATTGCAGCACCAGCGTTTG
cDNA amplification reverse primer (T7 terminator)	CTAGTTATTGCTCAGCGGT
Loop NNS and VNS NGS PCR1 forward primer	TCGTCGGCAGCGTCAGATGTGTATAAGAGACAGAATCTGGTACCACTT GGTCGC
Loop NNS and VNS NGS PCR1 reverse primer	GTCTCGTGGGCTCGGAGATGTGTATAAGAGACAGGGACAGGACAATT GTTTTCGAACC
C-term NNS and VNS NGS PCR1 forward primer	TCGTCGGCAGCGTCAGATGTGTATAAGAGACAGGGTGGCGCTAGCGA AAGC
C-term NNS and VNS NGS PCR1 reverse primer	GTCTCGTGGGCTCGGAGATGTGTATAAGAGACAGGTGGTGGTGCTCG CAATTGTTA

Table S2. Validation of assembled VLP variants from the NNS and VNS libraries.

Name	sequence	Assembled when expressed individually?	Freq. in G2 Library	Charge at pH 7.4	GRAVY value	Yield (mg/ L)
Loop NNS-1	TSSTREKSIGASTSL	Yes	2917	0	-0.6	6
Loop NNS-2	TAECHRGATNLLITV	Yes	296	-1.4	0.3	2
Loop NNS-3	LSGGTSDMRARRWRL	Yes	191	2	-0.9	0.2
Loop NNS-4	MSEETLEDRAEETSN	Yes	44	-5	-1.6	18.4
Loop NNS-5	ESSAAPSDKDLIDTP	Yes	40	-3	-0.8	45
Loop NNS-6	PTRPRSTSTVPSRMR	No	17	4	-1.4	-
Loop NNS-7	HPVRPRDRERIRARD	No	5	3	-2.2	-
Loop NNS-8	RTIRGRNSGRRHLGY	No	3	5	-1.7	-
Loop NNS-9	GSARSCSTCNVILCG	No	3	-0.3	0.7	-
Loop NNS-10	VTLFAAPSCIIKIIV	No	2	0.5	2.1	-
Loop VNS-1	IESNSTEVTSNVGIE	Yes	2000	-3	-0.3	18.9
Loop VNS-2	AVNPGAHNGINRQMR	Yes	684	2	-1.0	6
Loop VNS-3	MGKHGETPQHVNQRR	Yes	309	2	-2.0	6
Loop VNS-4	VPAAGPRTVPPAPSV	Yes	136	1	0.2	3.2
Loop VNS-5	HRMAHDTPTNNVNHI	Yes	105	0	-1.2	8
Loop VNS-6	GLTGRTVVQRKVAKK	No	65	4.9	-0.5	-
Loop VNS-7	PGRLVEVKALTGQAS	No	18	1	0.0	-
Loop VNS-8	VGKSNVALRHVVVLV	No	4	2	1.2	-
Loop VNS-9	IRANRTPPGARIHVD	Yes	3	2	-0.7	0.3
Loop VNS-10	QSRRPSETTAHRLRR	No	3	4	-2.1	-
C-term NNS-1	WSGEYQDNRISNKVA	Yes	442	-1	-1.3	>10*
C-term NNS-2	AGCFREERTISETED	Yes	131	-4.4	-1.2	>8*
C-term NNS-3	MPSKLRTTQVARTPA	Yes	35	2	-0.6	36
C-term NNS-4	RSRRTKSVERNWQRL	Yes	16	4	-2.1	>4*
C-term NNS-5	WPGEYQDNRISNKVA	No	7	-1	-1.4	-
C-term NNS-6	SSVMNQPYVGLCLLV	No	7	-1.4	1.1	-
C-term NNS-7	AWSEMVAGPVWASSI	No	3	-2	0.7	-
C-term NNS-8	GTEPDDEFAAWATPD	Yes	3	-6	-1.0	13.8
C-term NNS-9	VLLGYIWRLGVPAST	No	2	0	1.0	-
C-term NNS-10	ISQWPISFWRKDIWC	No	2	-0.5	-0.2	-
C-term VNS-1	NADDRVLGHKVPIVA	Yes	542	-1	0.0	0.4
C-term VNS-2	RPGRDTRGQAGGLER	Yes	154	1	-1.8	14.4
C-term VNS-3	VGELDATMQTLEEVG	Yes	47	-5	0.0	9.6
C-term VNS-4	MAPTSVGLTTEMLGT	No	47	-2	0.5	-
C-term VNS-5	QETHMNGTLAHPRGA	Yes	33	-1	-1.1	1.6
C-term VNS-6	QHKHKIRTDGTRAGS	Yes	26	2	-1.8	2.4
C-term VNS-7	RRRHGTTVNDPVTQG	Yes	7	2	-1.6	10.8
C-term VNS-8	RKGLRRHPDGKPRAM	No	4	4	-1.9	-
C-term VNS-9	VLGTDGVNLGSGVLV	No	2	-2	1.2	-
C-term VNS-10	QAPPEDGGGMTDDVV	Yes	2	-4	-0.7	6.9

* The purified VLP were partially insoluble, and only the concentration of the soluble fraction was measured.

Table S3. Log_2 (fold change) in frequency of each amino acid within the 15-mer positions from the isolated G1 loop NNS VLP library compared to its corresponding transformed plasmid library (Figure 4a). Minimum value in this table = -3.05 (8.3-fold reduced representation in the selected cDNA library relative to the starting transformed library); maximum value = 0.99 (2.0-fold enhanced representation in the selected cDNA library).

	A	G	I	L	M	P	V	N	Q	S	T	H	K	R	D	E	Y	F	W	C
**1**	0.49	0.40	-0.56	-0.72	-0.73	-0.03	-0.17	0.32	0.25	0.21	0.37	0.03	0.35	-0.07	0.99	0.74	-0.82	-1.44	-1.71	-0.77
**2**	0.25	0.33	-0.45	-0.76	-0.74	0.27	-0.34	0.28	0.37	0.18	0.16	0.28	0.48	-0.19	0.83	0.81	-0.93	-1.26	-2.38	-1.07
**3**	0.27	0.36	-0.86	-0.60	-0.57	0.17	-0.26	0.31	0.35	0.34	0.31	0.11	0.29	-0.13	0.78	0.68	-1.12	-1.63	-2.14	-1.21
**4**	0.28	0.41	-0.68	-0.76	-0.63	0.17	-0.33	0.37	0.40	0.29	0.22	0.27	0.29	-0.32	0.94	0.78	-1.34	-1.46	-2.44	-1.24
**5**	0.34	0.17	-0.60	-0.63	-0.32	0.29	-0.13	0.39	0.24	0.24	0.26	0.36	0.19	-0.25	0.80	0.75	-1.27	-1.77	-2.62	-1.53
**6**	0.31	0.26	-0.60	-0.67	-0.59	0.36	-0.11	0.47	0.20	0.30	0.17	0.08	0.41	-0.25	0.84	0.74	-1.21	-1.59	-3.02	-1.43
**7**	0.24	0.35	-0.51	-0.65	-0.74	0.24	-0.25	0.16	0.39	0.25	0.32	0.20	0.45	-0.39	0.86	0.74	-1.23	-1.60	-2.86	-1.61
**8**	0.33	0.31	-0.26	-0.80	-0.75	0.13	-0.17	0.36	0.38	0.33	0.35	0.03	0.37	-0.30	0.80	0.72	-1.68	-1.60	-3.05	-1.61
**9**	0.20	0.20	-0.51	-0.64	-0.57	0.28	0.01	0.40	0.28	0.29	0.30	0.22	0.37	-0.30	0.84	0.65	-1.06	-1.34	-2.36	-1.16
**10**	0.33	0.23	-0.42	-0.77	-0.40	0.30	-0.29	0.40	0.23	0.26	0.36	0.39	0.29	-0.37	0.80	0.53	-0.80	-1.16	-1.94	-1.05
**11**	0.11	0.34	-0.46	-0.56	-0.27	0.20	-0.28	0.44	0.18	0.26	0.24	0.24	0.24	-0.32	0.69	0.61	-0.83	-0.96	-1.48	-0.51
**12**	0.24	0.22	-0.33	-0.52	-0.22	0.07	-0.16	0.42	0.31	0.17	0.31	0.22	0.16	-0.25	0.54	0.61	-0.41	-1.08	-1.65	-0.50
**13**	0.21	0.17	-0.42	-0.38	-0.26	0.28	-0.25	0.26	0.20	0.16	0.20	0.20	0.14	-0.29	0.61	0.56	-0.28	-0.69	-0.59	-0.40
**14**	0.25	0.19	-0.23	-0.44	-0.36	0.19	-0.27	0.25	0.35	0.18	0.20	0.12	0.04	-0.36	0.59	0.56	-0.26	-0.69	-0.52	-0.32
**15**	0.22	0.04	-0.30	-0.30	0.01	0.23	-0.24	0.28	0.23	0.04	0.05	0.14	0.23	-0.09	0.34	0.70	-0.32	-0.73	-1.22	-0.36

Table S4. Log_2 (fold change) in frequency of each amino acid within the 15-mer positions from the isolated G1 C-term NNS VLP library compared to its corresponding transformed plasmid library (Figure 4b). Minimum value in this table = -4.99 (31.8-fold reduced representation in the selected cDNA library relative to the starting transformed library); maximum value = 0.87 (1.8-fold enhanced representation in the selected cDNA library).

	A	G	I	L	M	P	V	N	Q	S	T	H	K	R	D	E	Y	F	W	C
**1**	0.09	0.36	-0.56	-0.19	-0.08	0.10	-0.19	0.34	0.27	0.05	0.33	0.35	0.16	-0.27	0.59	0.62	0.01	-0.56	-1.28	-2.35
**2**	0.07	0.21	-0.39	-0.32	0.04	0.17	-0.08	0.19	0.31	0.12	0.44	0.10	-0.06	-0.15	0.50	0.67	-0.44	-0.91	-1.46	-2.80
**3**	0.31	0.12	0.05	-0.11	-0.21	0.27	-0.01	0.33	0.17	0.22	-0.23	0.09	-0.26	-0.17	0.54	0.60	-0.30	-0.68	-1.68	-2.72
**4**	0.32	0.29	-0.34	-0.42	-0.15	0.22	-0.07	-0.34	0.46	0.24	0.20	0.18	-0.11	-0.18	0.60	0.74	-0.67	-1.09	-1.18	-3.02
**5**	0.29	0.26	-0.44	-0.79	-0.07	0.38	-0.21	0.38	0.18	0.19	0.16	0.23	-0.13	-0.18	0.74	0.69	-0.04	-0.86	-1.75	-4.43
**6**	0.22	0.42	-0.93	-0.38	-0.63	0.22	0.03	0.41	0.15	0.23	0.35	0.08	-0.20	-0.39	0.76	0.71	-0.55	-1.16	-1.92	-3.54
**7**	0.14	0.26	-0.46	-0.47	-0.17	0.37	0.19	0.44	0.32	0.28	0.25	0.26	-0.01	-0.25	0.66	0.52	-1.06	-1.43	-2.88	-4.27
**8**	0.30	0.38	-0.38	-0.64	-0.37	0.51	0.10	0.28	0.07	0.14	0.30	-0.48	0.19	-0.28	0.57	0.64	-0.78	-1.13	-2.44	-3.61
**9**	0.22	0.21	-0.18	-0.55	-0.19	0.30	0.19	0.43	0.22	0.23	0.29	0.03	-0.03	-0.26	0.81	0.77	-0.87	-1.13	-2.94	-4.84
**10**	0.10	0.29	-0.16	-0.85	-0.28	0.31	0.11	0.54	0.24	0.28	0.28	0.39	-0.20	-0.41	0.56	0.87	-1.24	-1.26	-2.81	-3.46
**11**	0.43	0.15	-0.51	-0.34	-0.37	0.45	0.11	0.30	0.39	0.19	0.25	-0.01	0.12	-0.32	0.55	0.12	-0.66	-1.22	-2.63	-2.81
**12**	0.22	0.38	0.02	-0.38	-0.39	0.14	0.30	0.41	0.30	0.21	0.42	0.00	-0.26	-0.53	0.44	0.83	-0.77	-1.13	-2.70	-3.13
**13**	0.52	-0.01	0.13	-0.62	-0.11	0.22	0.28	0.43	0.14	0.30	0.32	0.26	0.31	-0.59	0.42	0.68	-0.88	-1.48	-2.46	-4.99
**14**	0.61	0.05	-0.14	-0.28	-0.42	0.35	0.39	0.17	0.37	0.26	-0.14	0.26	-0.36	-0.36	0.47	0.43	-0.68	-0.91	-2.10	-3.09
**15**	0.57	0.29	-0.84	-0.39	-0.25	0.26	-0.19	-0.13	0.33	0.50	0.19	0.01	0.12	-0.30	0.51	0.51	-1.76	-1.25	-3.87	-2.53

Table S5. Log$_2$ (fold change) in frequency of each amino acid within the 15-mer positions from the isolated G1 loop VNS VLP library compared to its corresponding transformed plasmid library (Figure 4c). Minimum value in this table = -0.84 (1.8-fold reduced representation in the selected cDNA library relative to the starting transformed library); maximum value = 0.90 (1.9-fold enhanced representation in the selected cDNA library).

	A	G	I	L	M	P	V	N	Q	S	T	H	K	R	D	E
**1**	0.36	0.23	-0.76	-0.60	-0.54	0.11	-0.22	0.24	0.26	0.12	0.08	0.20	-0.06	-0.38	0.81	0.74
**2**	0.12	0.18	-0.56	-0.67	-0.40	0.12	-0.27	0.21	0.34	0.16	0.14	-0.08	0.25	-0.43	0.61	0.66
**3**	0.13	0.18	-0.54	-0.54	-0.47	0.09	-0.40	0.25	0.17	0.19	0.08	0.30	0.20	-0.45	0.59	0.63
**4**	0.16	0.19	-0.53	-0.64	-0.53	0.12	-0.28	0.30	0.17	0.19	0.14	0.20	0.17	-0.48	0.56	0.62
**5**	0.24	0.16	-0.49	-0.84	-0.43	0.11	-0.15	0.18	0.17	0.03	0.13	0.17	0.24	-0.49	0.53	0.60
**6**	0.18	0.16	-0.44	-0.61	-0.59	0.11	-0.16	0.13	0.22	0.13	0.13	0.30	0.26	-0.55	0.65	0.60
**7**	0.11	0.14	-0.44	-0.62	-0.55	0.07	-0.20	0.23	0.25	0.03	0.18	0.08	0.27	-0.56	0.67	0.62
**8**	0.10	0.16	-0.52	-0.57	-0.54	0.06	-0.16	0.25	0.15	0.19	0.17	0.19	0.11	-0.43	0.60	0.55
**9**	0.13	0.16	-0.61	-0.47	-0.44	0.13	-0.20	0.25	0.27	0.03	0.15	0.30	0.00	-0.51	0.58	0.64
**10**	0.12	0.23	-0.69	-0.55	-0.27	0.06	-0.22	0.25	0.15	0.30	0.16	0.25	0.04	-0.41	0.46	0.42
**11**	-0.05	0.27	-0.47	-0.51	-0.28	0.05	-0.24	0.26	0.19	0.18	0.17	0.13	-0.04	-0.27	0.60	0.35
**12**	0.04	0.18	-0.42	-0.37	-0.13	0.07	-0.23	0.18	0.09	0.07	0.12	0.18	-0.05	-0.42	0.54	0.55
**13**	0.00	0.20	-0.23	-0.28	-0.15	-0.01	-0.25	0.26	0.21	0.15	0.10	0.22	-0.23	-0.47	0.67	0.59
**14**	0.25	0.07	-0.33	-0.58	-0.24	0.05	-0.33	0.30	0.18	0.13	0.10	0.09	-0.29	-0.29	0.76	0.79
**15**	0.10	-0.38	-0.77	-0.27	0.05	-0.08	-0.52	0.31	0.41	-0.29	-0.17	-0.01	0.09	-0.01	0.90	0.90

Table S6. Log$_2$ (fold change) in frequency of each amino acid within the 15-mer positions from the isolated G1 C-term VNS VLP library compared to its corresponding transformed plasmid library (Figure 4d). Minimum value in this table = -0.98 (2.0-fold reduced representation in the selected cDNA library relative to the starting transformed library); maximum value = 1.29 (2.5-fold enhanced representation in the selected cDNA library).

	A	G	I	L	M	P	V	N	Q	S	T	H	K	R	D	E
**1**	-0.13	0.07	-0.13	-0.14	-0.21	0.04	-0.19	0.12	0.29	0.05	0.03	0.04	-0.10	0.15	0.07	0.12
**2**	-0.11	0.00	-0.16	-0.26	-0.03	-0.04	-0.05	0.14	0.09	-0.03	-0.04	1.01	-0.19	-0.22	0.13	0.31
**3**	-0.07	-0.02	-0.44	-0.45	0.79	0.01	-0.15	0.21	0.06	0.17	0.05	0.12	-0.03	-0.28	0.11	0.37
**4**	0.03	0.08	-0.56	-0.48	-0.33	-0.01	-0.09	0.19	-0.10	0.18	0.08	1.01	-0.05	-0.33	0.27	0.34
**5**	-0.09	0.08	-0.40	-0.39	-0.34	0.02	-0.08	0.09	0.98	0.13	-0.03	0.08	-0.06	-0.36	0.33	0.37
**6**	-0.12	0.03	-0.28	-0.42	-0.10	0.13	-0.17	-0.12	0.17	0.21	-0.03	0.11	0.72	-0.32	0.28	0.41
**7**	0.07	0.10	-0.51	-0.62	-0.31	-0.04	-0.07	0.21	0.02	0.02	0.06	0.11	-0.11	-0.42	1.09	0.47
**8**	0.04	0.04	-0.43	-0.49	-0.28	0.13	-0.37	0.06	0.17	0.21	0.07	0.07	-0.17	-0.41	1.11	0.50
**9**	-0.04	0.05	-0.52	0.23	-0.38	0.07	-0.07	0.23	0.30	0.05	-0.09	0.19	-0.07	-0.50	0.34	0.58
**10**	0.11	0.06	-0.42	-0.38	-0.11	-0.02	-0.10	-0.01	-0.13	0.13	0.55	-0.05	-0.18	-0.49	0.41	0.52
**11**	0.00	0.01	-0.39	-0.43	-0.22	0.06	-0.16	-0.09	0.20	0.01	0.05	0.02	-0.17	-0.49	1.29	0.54
**12**	-0.03	0.06	-0.31	-0.46	-0.30	0.07	0.02	0.21	0.29	0.16	0.04	-0.10	0.75	-0.60	0.35	0.37
**13**	0.16	0.06	-0.41	-0.69	0.07	-0.06	0.45	-0.17	0.09	-0.02	0.15	-0.17	-0.27	-0.35	0.38	0.64
**14**	0.32	0.33	-0.84	-0.59	-0.27	-0.21	0.23	-0.38	0.95	0.08	-0.17	-0.37	-0.19	-0.35	0.15	0.69
**15**	0.81	0.23	-0.80	-0.98	-0.29	-0.46	-0.01	-0.28	0.21	0.20	0.44	-0.51	0.15	-0.46	-0.16	0.45

Table S7. Log_2 (fold change) in frequency of each amino acid within the 15-mer positions from the isolated G2 loop NNS VLP library compared to its corresponding transformed plasmid library (Figure S13a). Minimum value in this table = -3.42 (10.7-fold reduced representation in the selected cDNA library relative to the starting transformed library); maximum value = 2.12 (4.4-fold enhanced representation in the selected cDNA library).

	A	G	I	L	M	P	V	N	Q	S	T	H	K	R	D	E	Y	F	W	C
**1**	0.37	-0.21	0.16	-0.30	-0.06	-0.50	0.62	0.06	-0.41	-0.44	0.78	-0.70	0.11	-0.43	-0.15	0.32	0.14	-0.55	-0.96	-0.77
**2**	-0.30	0.08	-0.36	-0.10	-0.29	0.61	-0.80	0.19	-0.46	0.66	-0.23	-0.40	-0.10	-0.41	-0.19	0.26	-0.33	-1.31	-0.75	-0.67
**3**	-0.07	0.09	0.14	-0.49	0.53	-0.54	0.31	-0.68	-0.19	0.60	-0.30	-0.19	0.15	-0.08	-0.36	0.04	-0.05	0.37	1.50	-1.56
**4**	-0.21	0.12	-0.29	-0.12	-0.14	-0.15	-0.28	0.02	0.00	-0.53	1.09	-0.01	-0.33	-0.28	-0.14	0.32	-0.17	-0.21	0.03	-0.18
**5**	0.55	-0.49	0.27	-0.13	0.51	-0.33	0.29	-0.36	0.04	-0.64	-0.14	0.42	-0.43	0.70	-0.24	0.04	-1.12	-0.34	0.34	-0.45
**6**	-0.07	-0.09	-0.41	-0.17	-0.47	0.45	0.20	0.16	0.74	-0.27	-0.22	-0.28	-0.12	-0.47	-0.32	1.24	-0.26	-0.56	0.36	-1.35
**7**	0.22	0.09	0.50	-0.13	-0.18	0.24	-0.44	-0.45	-0.26	-0.28	-0.30	-0.13	1.04	-0.82	0.01	0.38	0.64	-0.11	-1.79	-1.20
**8**	-0.23	-0.21	1.57	-0.86	-0.20	0.05	-0.75	0.21	-0.28	0.62	-0.44	0.29	-0.05	-0.31	0.60	-0.34	-0.20	-1.08	-0.94	-1.69
**9**	-0.38	0.36	2.12	-0.36	0.16	-0.50	-0.33	0.14	-0.01	-0.06	0.42	-0.28	0.07	-0.38	-0.13	-0.41	0.57	-1.74	0.18	-3.42
**10**	-0.36	0.62	-0.51	-0.36	-0.20	-0.50	-0.11	0.79	0.17	-0.34	0.02	0.28	0.04	-0.38	0.25	-0.02	-0.16	0.12	0.04	-1.23
**11**	0.84	-0.36	-0.33	0.42	-0.69	-0.32	-0.22	0.01	-0.59	-0.11	-0.17	-0.16	-0.08	-0.27	-0.04	0.74	0.62	0.48	0.40	-0.50
**12**	0.12	-0.27	0.09	0.00	-0.17	-0.39	-0.11	0.01	-0.37	0.72	-0.26	0.31	-0.39	0.33	-0.14	-0.11	-0.50	-0.70	-0.23	-1.03
**13**	0.06	0.03	-0.66	-0.68	-0.38	-0.06	-0.33	0.17	-0.29	-0.42	0.94	-0.12	-0.11	-0.26	0.79	0.30	-0.03	-0.64	-0.04	-0.66
**14**	-0.20	-0.29	-1.19	-0.64	-1.09	0.05	-0.32	0.10	0.42	0.54	0.06	0.16	0.21	0.20	0.29	0.33	-0.68	-0.01	0.03	-1.61
**15**	-0.44	-0.62	-0.99	0.52	-0.43	-0.56	-0.65	0.32	0.17	-0.63	-0.59	-0.55	0.87	0.47	0.46	0.88	1.06	-0.79	-0.83	-1.06

Table S8. Log$_2$ (fold change) in frequency of each amino acid within the 15-mer positions from the isolated G2 C-term NNS VLP library compared to its corresponding transformed plasmid library (Figure S13b). Minimum value in this table = -5.02 (32.4-fold reduced representation in the selected cDNA library relative to the starting transformed library); maximum value = 2.22 (4.7-fold enhanced representation in the selected cDNA library).

	A	G	I	L	M	P	V	N	Q	S	T	H	K	R	D	E	Y	F	W	C
**1**	0.05	0.13	-0.32	1.62	-0.53	-0.73	-0.49	-0.40	-0.30	-0.25	-0.81	-0.20	0.02	0.64	-0.51	-0.20	0.37	-0.52	1.06	-1.79
**2**	-0.43	-0.11	-0.21	0.86	-0.31	0.68	-0.09	-0.67	-0.51	0.21	0.03	-0.36	-0.85	-0.29	-1.18	0.60	0.13	-0.30	-0.66	-0.52
**3**	-0.93	0.22	0.36	-0.13	-0.38	-0.45	-0.52	-1.32	-0.20	0.91	-0.66	2.22	-0.25	-0.05	-0.27	-0.78	-1.57	-0.39	-0.85	-0.04
**4**	-0.57	0.27	1.57	0.09	-1.17	-0.65	-0.34	-0.17	-0.33	-0.32	0.15	-0.70	-0.71	0.44	-0.04	0.86	-0.07	-0.54	0.31	-0.48
**5**	0.54	-0.16	-0.13	-0.56	0.34	-0.31	-0.08	-0.64	0.03	0.19	0.04	0.21	-1.04	0.19	-0.51	-0.23	1.75	-0.69	0.70	-0.67
**6**	-1.29	-0.26	-0.29	-0.25	0.37	-0.03	-0.99	-0.30	0.70	-0.05	1.01	0.20	0.16	0.71	-0.61	0.12	-1.20	0.24	-0.77	-1.50
**7**	-0.44	-0.36	-1.03	0.01	-0.32	0.71	0.34	0.28	-0.53	0.23	-0.58	-0.78	0.64	-0.43	0.35	0.05	-0.16	-1.10	-0.67	0.56
**8**	0.11	-0.22	0.35	0.48	-0.27	-0.37	0.13	0.44	-0.58	0.37	-0.54	0.96	-0.36	-0.22	-0.91	-0.18	0.38	-0.86	-1.18	-1.52
**9**	-1.00	0.14	0.68	-0.50	-0.96	-0.24	0.11	-0.53	1.30	-0.69	-0.21	-0.45	-0.54	0.04	1.13	-0.14	-0.81	-0.92	-0.03	-1.36
**10**	0.31	0.11	1.75	0.02	-0.21	-0.28	-0.24	-0.91	0.00	-0.33	1.05	-0.86	-0.13	-0.47	0.71	-0.58	-1.53	-0.93	-3.40	-1.90
**11**	0.98	0.24	-1.27	-0.51	-0.59	-0.44	0.59	-0.56	0.07	0.29	-0.47	-0.20	-0.60	-0.35	-0.02	-0.42	-1.38	0.18	-0.57	-3.42
**12**	0.20	-0.63	-0.76	0.18	-0.81	-0.95	-0.11	1.78	-0.25	-0.14	-0.73	0.08	-0.31	-0.54	0.84	0.24	0.63	-0.18	1.22	-0.05
**13**	-0.63	0.04	-1.20	-0.39	-0.17	0.12	-0.25	-0.02	0.26	0.07	0.10	-0.78	0.95	0.18	-1.45	1.26	0.47	-0.92	-2.61	-0.62
**14**	0.35	0.60	-0.70	-0.83	-0.69	-0.49	0.93	-1.35	-0.59	-0.24	0.22	-0.89	-1.19	0.22	-0.44	0.19	0.06	-1.28	-1.04	-5.02
**15**	0.28	-0.63	0.44	-0.12	0.14	-0.85	-0.22	-0.65	0.65	0.78	0.06	-0.66	1.51	-0.77	-0.70	-0.81	0.01	-1.40	-0.78	0.26

Table S9. Log_2 (fold change) in frequency of each amino acid within the 15-mer positions from the isolated G2 loop VNS VLP library compared to its corresponding transformed plasmid library (Figure S13c). Minimum value in this table = -0.77 (1.7-fold reduced representation in the selected cDNA library relative to the starting transformed library); maximum value = 1.39 (2.6-fold enhanced representation in the selected cDNA library).

	A	G	I	L	M	P	V	N	Q	S	T	H	K	R	D	E
**1**	0.51	-0.03	-0.07	-0.42	-0.14	-0.21	0.32	-0.46	-0.22	-0.20	-0.34	-0.31	-0.05	-0.40	0.67	0.37
**2**	-0.22	-0.03	-0.13	-0.18	-0.10	-0.07	-0.12	-0.16	0.57	-0.27	0.01	-0.61	0.03	-0.28	0.09	0.76
**3**	-0.12	0.16	-0.52	-0.21	-0.24	-0.05	-0.41	-0.06	-0.52	0.23	-0.01	0.64	0.43	-0.35	0.64	-0.30
**4**	-0.13	0.15	0.47	-0.37	-0.21	-0.21	0.07	0.08	0.00	-0.22	0.09	0.12	0.42	-0.35	0.09	0.14
**5**	0.42	-0.07	-0.06	-0.42	-0.57	-0.08	0.02	0.18	-0.07	0.17	-0.09	-0.12	0.01	-0.30	-0.09	0.52
**6**	-0.11	-0.05	-0.18	-0.20	-0.40	-0.02	0.40	-0.27	-0.22	0.07	0.07	-0.13	0.27	-0.01	0.11	0.01
**7**	0.31	-0.44	-0.37	0.00	0.06	0.19	-0.33	1.04	-0.06	0.23	-0.11	-0.49	-0.13	-0.34	0.00	0.18
**8**	0.12	-0.09	-0.34	-0.30	0.22	-0.36	0.31	0.35	-0.24	-0.23	0.18	0.09	-0.41	-0.40	0.52	-0.08
**9**	-0.02	-0.14	0.70	-0.05	-0.22	-0.05	0.01	-0.44	0.65	0.06	-0.18	-0.20	0.34	-0.35	0.14	0.08
**10**	-0.05	0.12	0.04	-0.01	0.07	-0.17	0.10	-0.25	-0.28	0.11	-0.09	-0.19	0.38	-0.14	0.00	0.28
**11**	0.46	0.21	-0.20	-0.05	-0.55	0.12	-0.07	0.14	-0.03	-0.42	-0.14	0.13	-0.48	-0.08	0.14	-0.12
**12**	0.00	-0.20	-0.12	-0.06	0.33	0.43	0.01	-0.05	0.20	-0.39	0.00	0.09	-0.14	-0.05	0.08	-0.25
**13**	-0.31	0.07	-0.27	0.45	-0.40	-0.32	-0.14	0.39	0.36	-0.13	-0.48	0.08	0.44	-0.40	0.27	0.35
**14**	0.03	-0.21	-0.01	-0.16	0.28	-0.40	-0.01	-0.04	-0.05	0.00	-0.06	-0.25	0.15	-0.38	0.54	0.75
**15**	-0.02	-0.51	-0.66	-0.52	-0.28	-0.62	-0.59	-0.37	0.33	-0.65	-0.77	-0.62	0.25	-0.07	1.18	1.39

Table S10. Log_2 (fold change) in frequency of each amino acid within the 15-mer positions from the isolated G2 C-term VNS VLP library compared to its corresponding transformed plasmid library (Figure S13d). Minimum value in this table = -0.99 (2-fold reduced representation in the selected cDNA library relative to the starting transformed library); maximum value = 1.11 (2.2-fold enhanced representation in the selected cDNA library).

	A	G	I	L	M	P	V	N	Q	S	T	H	K	R	D	E
**1**	-0.14	-0.18	-0.04	-0.31	0.12	0.17	-0.06	0.35	0.44	-0.51	-0.22	-0.17	-0.28	0.02	-0.26	0.53
**2**	0.28	0.00	0.17	0.29	0.36	0.02	-0.13	-0.04	-0.31	-0.28	-0.10	0.12	-0.37	-0.11	-0.20	-0.32
**3**	-0.09	0.32	-0.43	-0.30	0.20	0.14	-0.25	0.33	0.13	-0.21	-0.42	-0.21	-0.10	0.24	-0.07	-0.03
**4**	-0.41	-0.03	-0.46	0.26	0.29	0.03	0.05	-0.22	0.00	0.37	-0.23	0.01	0.16	-0.05	0.35	-0.01
**5**	0.01	-0.02	-0.42	-0.12	0.65	-0.24	0.04	-0.53	0.09	0.11	-0.01	-0.38	-0.03	0.27	-0.37	0.33
**6**	0.17	0.13	-0.40	-0.37	0.29	-0.34	0.13	-0.43	0.64	-0.25	0.15	-0.98	-0.04	0.12	-0.66	0.82
**7**	0.02	-0.48	0.16	0.05	-0.35	0.24	0.11	0.45	-0.55	0.40	-0.48	0.18	0.45	0.52	-0.27	-0.74
**8**	-0.16	0.04	1.11	0.37	-0.19	-0.89	0.10	-0.06	0.76	0.05	0.15	0.42	-0.21	-0.24	0.04	-0.12
**9**	-0.09	0.41	-0.99	-0.78	-0.38	0.55	0.11	-0.70	-0.39	0.22	0.49	-0.07	-0.60	-0.38	0.14	0.20
**10**	-0.25	0.21	-0.50	0.76	-0.07	0.21	-0.10	0.22	0.14	-0.04	-0.94	0.72	0.36	-0.39	0.60	-0.38
**11**	0.06	0.48	-0.32	-0.40	0.91	-0.53	-0.48	-0.13	0.92	-0.08	0.57	-0.25	-0.69	-0.01	-0.92	0.02
**12**	0.05	-0.29	-0.56	-0.44	-0.14	-0.18	0.56	-0.56	-0.52	0.07	0.40	-0.31	0.07	-0.20	0.55	0.52
**13**	0.22	-0.02	0.85	0.24	-0.05	0.59	-0.46	-0.41	-0.13	0.58	-0.48	-0.44	0.91	-0.71	0.19	-0.22
**14**	-0.39	-0.88	0.44	0.06	0.99	0.12	0.43	0.57	-0.62	-0.09	0.02	0.50	-0.82	0.02	0.47	0.15
**15**	0.16	-0.28	0.57	0.33	-0.38	0.02	0.71	-0.11	0.39	0.77	-0.43	0.25	-0.91	-0.52	-0.11	-0.12

References

1. Suloway, C.; Pulokas, J.; Fellmann, D.; Cheng, A.; Guerra, F.; Quispe, J.D.; Stagg, S.; Potter, C.S.; Carragher, B., Automated Molecular Microscopy: The new Leginon System. *J. Struct. Biol.* **2005**, *151*, 41-60. https://doi.org/10.1016/j.jsb.2005.03.010
2. Pettersen, E.F.; Goddard, T.D.; Huang, C.C.; Couch, G.S.; Greenblatt, D.M.; Meng, E.C.; Ferrin, T.E., UCSF Chimera—a visualization system for exploratory research and analysis. *J. Comput. Chem.* **2004**, *25*, 1605-1612. http://dx.doi.org/10.1002/jcc.20084
